# Genome-wide meta-analyses of restless legs syndrome yield insights into genetic architecture, disease biology and risk prediction

**DOI:** 10.1038/s41588-024-01763-1

**Published:** 2024-06-05

**Authors:** Barbara Schormair, Chen Zhao, Steven Bell, Maria Didriksen, Muhammad S. Nawaz, Nathalie Schandra, Ambra Stefani, Birgit Högl, Yves Dauvilliers, Cornelius G. Bachmann, David Kemlink, Karel Sonka, Walter Paulus, Claudia Trenkwalder, Wolfgang H. Oertel, Magdolna Hornyak, Maris Teder-Laving, Andres Metspalu, Georgios M. Hadjigeorgiou, Olli Polo, Ingo Fietze, Owen A. Ross, Zbigniew K. Wszolek, Abubaker Ibrahim, Melanie Bergmann, Volker Kittke, Philip Harrer, Joseph Dowsett, Sofiene Chenini, Sisse Rye Ostrowski, Erik Sørensen, Christian Erikstrup, Ole B. Pedersen, Mie Topholm Bruun, Kaspar R. Nielsen, Adam S. Butterworth, Nicole Soranzo, Willem H. Ouwehand, David J. Roberts, John Danesh, Brendan Burchell, Nicholas A. Furlotte, Priyanka Nandakumar, 23andMe Research Team, D.E.S.I.R. study group, Amélie Bonnefond, Louis Potier, Christopher J. Earley, William G. Ondo, Lan Xiong, Alex Desautels, Markus Perola, Pavel Vodicka, Christian Dina, Monika Stoll, Andre Franke, Wolfgang Lieb, Alexandre F. R. Stewart, Svati H. Shah, Christian Gieger, Annette Peters, David B. Rye, Guy A. Rouleau, Klaus Berger, Hreinn Stefansson, Henrik Ullum, Kari Stefansson, David A. Hinds, Emanuele Di Angelantonio, Konrad Oexle, Juliane Winkelmann

**Affiliations:** 1 Institute of Neurogenomics, Helmholtz Zentrum München, German Research Center for Environmental Health, Neuherberg, Germany; 2 Institute of Human Genetics, TUM School of Medicine and Health, Technical University of Munich, Munich, Germany; 3 Department of Oncology, University of Cambridge, Cambridge, UK; 4 Department of Clinical Neurosciences, University of Cambridge, Cambridge, UK; 5 Cancer Research UK Cambridge Institute, Li Ka Shing Centre, University of Cambridge, Cambridge, UK; 6 Department of Clinical Immunology, Copenhagen University Hospital, Rigshospitalet, Copenhagen, Denmark; 7 Department of Neuroscience, University of Copenhagen, Copenhagen, Denmark; 8 deCODE Genetics/Amgen, Reykjavik, Iceland; 9 Sleep Disorders Clinic, Department of Neurology, Medical University of Innsbruck, Innsbruck, Austria; 10 Sleep–Wake Disorders Center, Department of Neurology, Hôpital Gui-de-Chauliac, CHU Montpellier, Institut des Neurosciences de Montpellier, INSERM, Université de Montpellier, Montpellier, France; 11 SomnoDiagnostics, Osnabrück, Germany; 12 Department of Neurology, University Medical Center Göttingen, Göttingen, Germany; 13 Department of Neurology and Centre of Clinical Neuroscience, Charles University, First Faculty of Medicine and General University Hospital, Prague, Czech Republic; 14 Department of Neurology, Ludwig Maximilians University Munich, Munich, Germany; 15 Paracelsus-Elena-Klinik, Kassel, Germany; 16 Department of Neurosurgery, University Medical Center Göttingen, Göttingen, Germany; 17 Department of Neurology, Philipps-University Marburg, Marburg, Germany; 18 Neuropsychiatry Centre Erding/München, Erding, Germany; 19 Estonian Genome Center, Institute of Genomics, University of Tartu, Tartu, Estonia; 20 Department of Neurology, Nicosia General Hospital Medical School, University of Cyprus, Nicosia, Cyprus; 21 Bragée ME/CFS Center, Stockholm, Sweden; 22 Department of Pulmonology, Center of Sleep Medicine, Charité—Universitätsmedizin Berlin, Berlin, Germany; 23 Department of Neuroscience, Mayo Clinic College of Medicine, Jacksonville, FL USA; 24 Department of Neurology, Mayo Clinic, Jacksonville, FL USA; 25 Department of Clinical Medicine, University of Copenhagen, Copenhagen, Denmark; 26 Department of Clinical Immunology, Aarhus University Hospital, Aarhus, Denmark; 27 Department of Clinical Medicine, Aarhus University, Aarhus, Denmark; 28 Department of Clinical Immunology, Zealand University Hospital, Køge, Denmark; 29 Department of Clinical Immunology, Odense University Hospital, Odense, Denmark; 30 Department of Clinical Immunology, Aalborg University Hospital, Aalborg, Denmark; 31 British Heart Foundation Cardiovascular Epidemiology Unit, Department of Public Health and Primary Care, University of Cambridge, Cambridge, UK; 32 British Heart Foundation Centre of Research Excellence, University of Cambridge, Cambridge, UK; 33 National Institute for Health and Care Research Blood and Transplant Research Unit in Donor Health and Behaviour, University of Cambridge, Cambridge, UK; 34 Health Data Research UK Cambridge, Wellcome Genome Campus and University of Cambridge, Cambridge, UK; 35 Victor Phillip Dahdaleh Heart and Lung Research Institute, University of Cambridge, Cambridge, UK; 36 Department of Haematology, University of Cambridge, Cambridge, UK; 37 Department of Human Genetics, the Wellcome Trust Sanger Institute, Wellcome Trust Genome Campus, Hinxton, UK; 38 NHS Blood and Transplant, Cambridge Biomedical Campus, Cambridge, UK; 39 Department of Haematology, University College London Hospitals, London, UK; 40 Radcliffe Department of Medicine and National Health Service Blood and Transplant, Oxford, UK; 41 Department of Haematology and BRC Haematology Theme, Churchill Hospital, Headington, Oxford, UK; 42 Magdalene College, Cambridge, UK; 43 23andMe, Inc., Sunnyvale, CA USA; 44 Center for Restless Legs Syndrome, Department of Neurology, Johns Hopkins University, Baltimore, MD USA; 45 Department of Neurology, Methodist Neurological Institute, Weill Cornell Medical School, Houston, TX USA; 46 The Neuro (Montreal Neurological Institute–Hospital), McGill University, Montreal, Quebec Canada; 47 Department of Neurology and Neurosurgery, McGill University, Montreal, Quebec Canada; 48 Centre d’Études Avancées en Médecine du Sommeil, Hôpital du Sacré-Cœur de Montréal, Montreal, Quebec Canada; 49 Department of Neurosciences, Université de Montréal, Montreal, Quebec Canada; 50 Clinical and Molecular Metabolism Research Program (CAMM), Faculty of Medicine, University of Helsinki, Helsinki, Finland; 51 Department of Public Health and Welfare, National Institute for Health and Welfare, Helsinki, Finland; 52 Department of Molecular Biology of Cancer, Institute of Experimental Medicine, Academy of Science of Czech Republic, Prague, Czech Republic; 53 First Faculty of Medicine, Charles University in Prague, Prague, Czech Republic; 54 Biomedical Centre, Faculty of Medicine in Pilsen, Charles University in Prague, Pilsen, Czech Republic; 55 L’institut du thorax, CNRS, INSERM, Nantes Université, Nantes, France; 56 Department of Genetic Epidemiology, Institute for Human Genetics, University of Münster, Münster, Germany; 57 Institute of Clinical Molecular Biology, Kiel University, Kiel, Germany; 58 PopGen Biobank and Institute of Epidemiology, Christian Albrechts University Kiel, Kiel, Germany; 59 John and Jennifer Ruddy Canadian Cardiovascular Genetics Centre, University of Ottawa Heart Institute, Ottawa, Ontario Canada; 60 Department of Medicine, Duke University School of Medicine, Durham, NC USA; 61 Duke Clinical Research Institute, Duke University School of Medicine, Durham, NC USA; 62 Institute of Epidemiology, Helmholtz Zentrum München, German Research Center for Environmental Health, Neuherberg, Germany; 63 Research Unit of Molecular Epidemiology, Helmholtz Zentrum München, German Research Center for Environmental Health, Neuherberg, Germany; 64 German Research Center for Cardiovascular Disease (DZHK), partner site Munich Heart Alliance, Hannover, Germany; 65 Chair of Epidemiology, Institute for Medical Information Processing, Biometry and Epidemiology, Medical Faculty, Ludwig-Maximilians-Universität München, Munich, Germany; 66 Department of Neurology, Emory University, Atlanta, GA USA; 67 Department of Human Genetics, McGill University, Montreal, Quebec Canada; 68 Institute of Epidemiology and Social Medicine, University of Münster, Münster, Germany; 69 Statens Serum Institute, Copenhagen, Denmark; 70 Health Data Science Research Centre, Fondazione Human Technopole, Milan, Italy; 71 Neurogenetic Systems Analysis Group, Institute of Neurogenomics, Helmholtz Zentrum München, German Research Center for Environmental Health, Neuherberg, Germany; 72 Munich Cluster for Systems Neurology (SyNergy), Munich, Germany; 73 German Center for Mental Health (DZPG), partner site Munich–Augsburg, Munich–Augsburg, Germany; 74 Inserm U1283, CNRS UMR 8199, European Genomic Institute for Diabetes, Institut Pasteur de Lille, Lille, France; 75 University of Lille, Lille University Hospital, Lille, France; 76 Institut Necker-Enfants Malades, INSERM UMR-S1151, CNRS UMR-S8253, Université Paris Cité, Paris, France; 77 Department of Diabetology, Endocrinology and Nutrition, DHU FIRE, Assistance Publique-Hôpitaux de Paris, Bichat Hospital, Paris, France

**Keywords:** Genome-wide association studies, Genetics research, Sleep disorders

## Abstract

Restless legs syndrome (RLS) affects up to 10% of older adults. Their healthcare is impeded by delayed diagnosis and insufficient treatment. To advance disease prediction and find new entry points for therapy, we performed meta-analyses of genome-wide association studies in 116,647 individuals with RLS (cases) and 1,546,466 controls of European ancestry. The pooled analysis increased the number of risk loci eightfold to 164, including three on chromosome X. Sex-specific meta-analyses revealed largely overlapping genetic predispositions of the sexes (*r*_g_ = 0.96). Locus annotation prioritized druggable genes such as glutamate receptors 1 and 4, and Mendelian randomization indicated RLS as a causal risk factor for diabetes. Machine learning approaches combining genetic and nongenetic information performed best in risk prediction (area under the curve (AUC) = 0.82–0.91). In summary, we identified targets for drug development and repurposing, prioritized potential causal relationships between RLS and relevant comorbidities and risk factors for follow-up and provided evidence that nonlinear interactions are likely relevant to RLS risk prediction.

## Abstract (web summary)

Pooled and sex-specific genome-wide association analyses identify new risk loci for restless legs syndrome and candidates for drug repurposing. Machine learning models combining genetic and other information show improved risk prediction performance.

## Main

RLS is a prevalent, but underdiagnosed, chronic sensorimotor disorder, affecting up to 10% of the elderly population in Europe and North America^1,2^. Previous genome-wide association studies (GWAS) have identified 22 risk loci^3,4^. However, objective biomarkers for prediction or diagnosis are not available yet. Severely impairing sleep, RLS has a profound impact on daily functioning, overall health and quality of life. Long-term treatment options are scarce and require frequent adjustment due to side effects^2,5^.

RLS is often comorbid with psychiatric disorders such as depression or anxiety as well as cardiovascular disorders, hypertension and metabolic conditions such as diabetes^2,6^. The extent to which these associations imply causal relations is unknown^7^. Epidemiological and clinical studies have consistently demonstrated the prevalence of RLS to be twice as high in women than in men^8,9^. The contribution of genetic factors to this difference has not been examined yet.

To address these shortcomings, we conducted a genome-wide association meta-analysis (GWAMA) of three independent GWAS. We integrated multiple layers of functional omics data to identify pathways and cell types relevant to RLS. Furthermore, our analyses included sex-stratified GWAS and a genetic investigation of the X chromosome. To facilitate translational research, we identified drug targets among candidate genes, used machine learning to enhance risk prediction and conducted extensive genetic correlation and Mendelian randomization (MR) analyses to identify risk factors.

## Results

### Pooled autosomal GWAS meta-analysis

**Extended Data Fig. 1:**
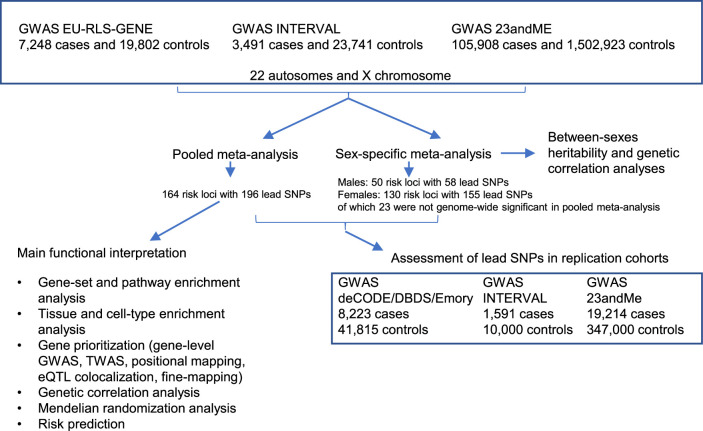
General study workflow. Overview of the main analytical steps conducted in the study. While sex-specific GWAS meta-analysis results were used to dissect similarities and differences between both sexes, the pooled meta-analysis results were used for further functional interpretation.

**Extended Data Fig. 2:**
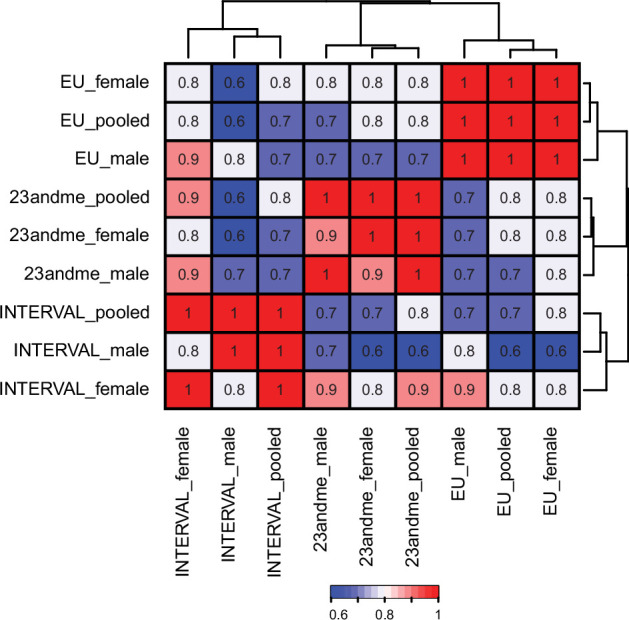
Genetic correlation between individual discovery stage GWAS of the N-GWAMA meta-analysis. Genetic correlations between the discovery stage input GWAS were calculated using LDSC on the summary statistics.

**Extended Data Fig. 3:**
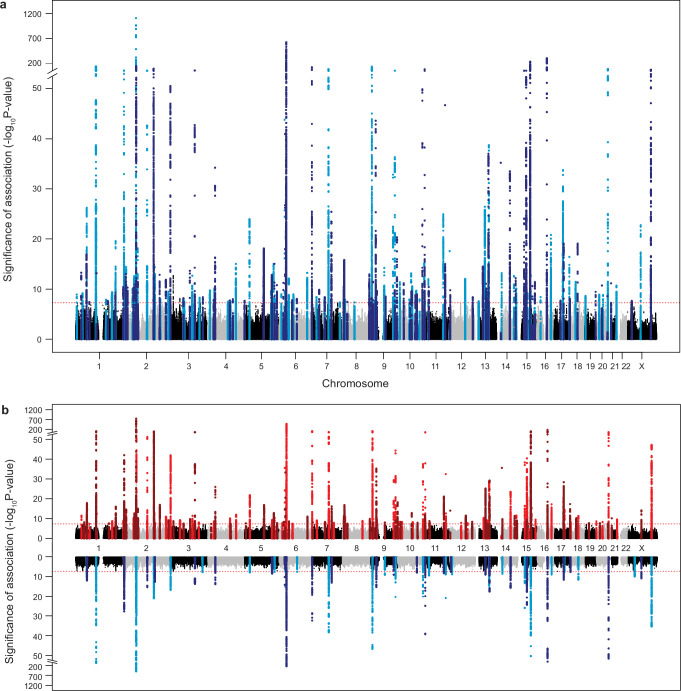
Manhattan and Miami plots of discovery stage meta-analyses. **a**, Results of the pooled discovery meta-analysis. **b**, Results of the sex-specific discovery meta-analyses. Female-only results are depicted in red in the upper section of the Miami plot, male-only results are depicted in blue in lower section of the Miami plot. The *x*-axis shows chromosome and base pair positions of the tested variants. The *y*-axis shows significance as −log_10_ of the two-sided nominal P-values of the *N*-GWAMA analyses. Red horizontal dashed lines indicate the Bonferroni-adjusted significant threshold of *P* < 5 × 10^−8^.

We performed a meta-analysis of summary statistics from three GWAS for RLS, totaling 116,647 cases and 1,546,466 controls of European ancestry (Extended Data Fig. 1). The first GWAS (EU-RLS-GENE) was conducted in affected individuals recruited by expert clinicians of the International EU-RLS-GENE consortium and ancestry-matched controls. The second GWAS (INTERVAL) was based on the INTERVAL study of blood donors in the United Kingdom, which used the Cambridge-Hopkins questionnaire to diagnose RLS. The third GWAS (23andMe) was conducted on the research participant base of 23andMe, identifying RLS by asking whether a diagnosis or treatment of RLS was received from a physician. Further details are provided in the Methods. Genetic correlations between the GWAS were strong but indicated some degree of heterogeneity, with pairwise genetic correlation (*r*_g_) ranging between 0.70 and 0.76 (Extended Data Fig. 2), possibly due to differences in phenotyping of RLS as well as in source populations targeted for recruitment. Therefore, we used a multivariate GWAMA approach (Methods). After quality control, 9,196,648 variants with minor allele frequency (MAF) ≥ 1% were available for meta-analysis. We identified 161 RLS risk loci (*P* < 5 × 10^−8^) on the autosomes, confirming all known loci and adding 139 new loci (Extended Data Fig. 3a). Conditional analysis within each locus resulted in a total of 193 independent lead SNPs (Supplementary Table 1).

An LD score regression (LDSC) intercept of 1.072 (standard error (s.e.) = 0.013) with an inflation ratio of 0.064 (s.e. = 0.012) indicated that population stratification was negligible and that the inflation of the test statistics was driven by the polygenic architecture of RLS.

At the meta-analysis level, assuming a disease prevalence of 9%, the overall SNP-based heritability was estimated to be 0.20 (s.e. = 0.016) using LDSC (Methods). Because the meta-analysis included studies with different phenotyping methods, we also derived heritability estimates from the individual GWAS. LDSC-derived heritability in the most stringently phenotyped study, EU-RLS-GENE, was higher (0.26, s.e. = 0.038) than that in INTERVAL (0.17, s.e. = 0.051, *P*_EU-Interval_ = 0.073, two-sample two-sided *Z*-test) and 23andMe (0.14, s.e. = 0.011, *P*_EU-23andMe_ = 0.0012, two-sample two-sided *Z*-test). While the LDSC model showed the best fit, this trend was consistent with other estimation methods (Supplementary Table 2a).

### Sex-stratified autosomal GWAS and meta-analyses

To study sex-specific genetic effects, we conducted sex-stratified GWAS for the autosomes in each study and meta-analyzed the results (Extended Data Fig. 3b, representing 78,333 cases and 844,872 controls in women and 38,314 cases and 701,594 controls in men). Heritability was significantly higher for females in the meta-analysis (${h}_{{\rm{males}}}^{2}= 0.13$, s.e. = 0.012; ${h}_{{\rm{females}}}^{2}=0.32$, s.e. = 0.027; *P*_difference_ = 1.9 × 10^−8^, two-sample two-sided *Z*-test). The INTERVAL study was too small for reliable application of LDSC, but both other cohorts showed higher estimates for LDSC-derived heritability in females than in males (*P*_difference_ = 0.07 in EU-RLS-GENE; *P*_difference_ = 0.09 in 23andMe, two-sample two-sided *Z*-test; Supplementary Table 2b,c). Comparing the two sex-specific meta-analyses showed a high genetic correlation of 0.96 (s.e. = 0.018); however, the remaining small divergence was significant (*P* = 0.044, one-sample two-sided *Z*-test).

The sex-specific meta-analyses identified 58 independent lead SNPs in 50 risk loci in males and 155 SNPs in 130 loci in females (Supplementary Tables 3 and 4). Of these loci, 23 (two in males, 21 in females) were not genome-wide significant in the pooled analysis. To prioritize loci with robust sex differences, we tested the lead SNPs of the pooled meta-analysis for heterogeneity of effect sizes between males and females. This was statistically significant for six loci (Extended Data Table 1).

**Extended Data Fig. 4:**
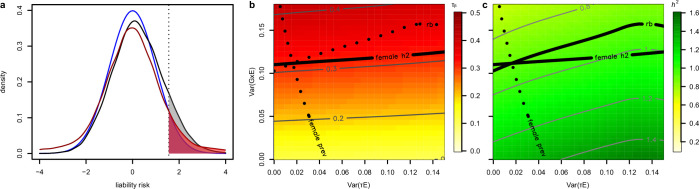
Simulation study assessing sex-specific heritability and genetic correlation divergence. Simulation of environmental effect that reconciles sex-difference in heritability with the similarity of the SNP effect sizes. **a**, Frequency density distributions of the liabilities for different models. Blue line, base model, $\varphi =X\beta +\varepsilon$, as assumed to be present in males with *h*^2^ = 0.1395, *X* and *β* as determined by GWAS, $\varepsilon \sim N(0,1)$, and a disease threshold in keeping with the male RLS prevalence of 0.06 (shaded area under the curve). Black line, model with non-interacting binary environmental effect, $\varphi =X\beta +\tau E+\varepsilon$, with $X,\beta ,\varepsilon$ and threshold as in the base model plus an additional binary effect $E \sim Bernoulli(p=0.21)$, representing childlessness with a weight vector *τ* such that that prevalence is 0.13 as in females. Red line, analogous *G*×*E* model, $\varphi =X\beta +X\eta \circ E+\varepsilon$, but where the environmental effect now interacts with the genetic effects and the corresponding weight vector *η* is chosen in accordance with the female prevalence. **b**, **c**, Optimization of the model $\varphi =X\beta +X\eta \circ E+\tau E+\varepsilon$ with $X,\beta ,E,\varepsilon$ and threshold as above, where the additional degree of freedom is covered by also considering the mean effect size ratio *rb* observed in the GWAS. Heatmap and contour plot for logistic regression-based liability scaled LDSC *h*^2^ (**b**) and effect size ratio *rb* (**c**) as functions of $Var(\tau E)$ and $Var(X\eta \circ E)$. Optimal values for $Var(\tau E)$ and $Var(X\eta \circ E)$, that is, for *τ* and *η*, respectively, comply with female prevalence, female heritability, and observed effect size ratio as well. The optimal *τ* turns out to be close to zero so that the environmental factor acts mostly via genetic interaction.

To understand the discrepancy between the heritability estimates of the two sexes despite their high genetic correlation, we ran a simulation study (Supplementary Note) modeling the impact of an environmental risk factor and of its interaction with the genetic predisposition to RLS (*G* × *E*). The results obtained with the model including the *G* × *E* interaction recapitulated the situation observed in our real-world GWAS data very closely. This was the case for both binary and continuous environmental factors, with the binary risk factor showing a slightly better fit (log_10_ (Bayes factor) of 11.43 compared to 9.11). In line with this, the *G* × *E* model showed a closer fit (*P* = 0.02, two-sample two-sided *Z*-test) to the ${h}_{\rm{male}}^{2}/{h}_{\rm{female}}^{2}$ ratio observed in the pooled GWAS than the model without the *G* × *E* interaction (Extended Data Fig. 4). Furthermore, the impact of a *G* × *E* interaction on RLS was higher in females than in males with a ${r}_{G\times E(\rm{female})}/{r}_{G\times E(\rm{male})}$ ratio of 16.1 (95% CI = 7.09, 51.12).

### X-chromosomal meta-analyses

**Extended Data Fig. 5:**
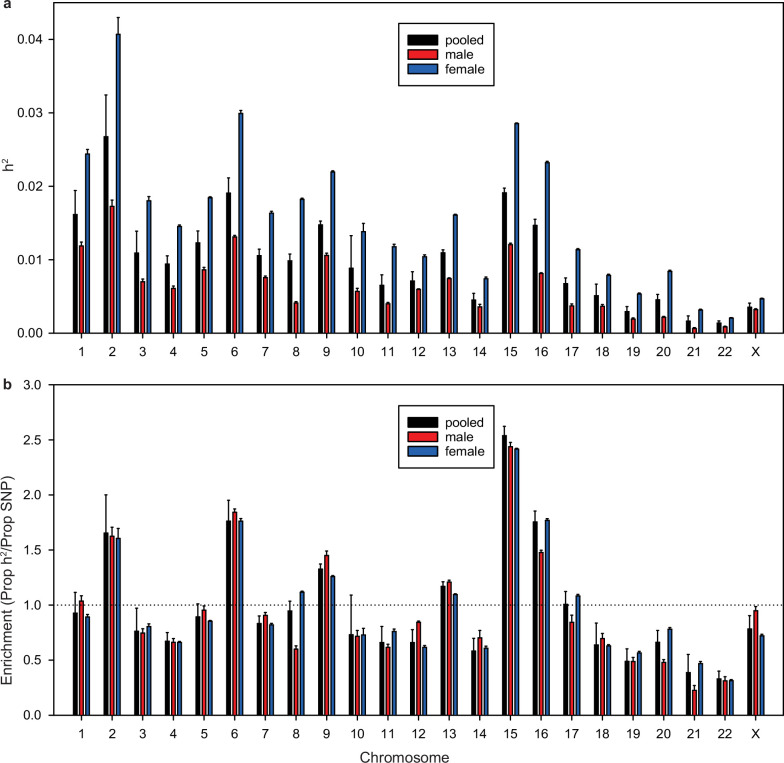
Per chromosome heritability estimation based on the EU-RLS-GENE dataset. Heritability estimates for each chromosome. **a**, Overall heritability of SNPs on each chromosome. The height of the bar represents the point estimate of the heritability, and the error bars indicate the standard error of this point estimate. **b**, Enrichment of heritability, which is defined as the proportion of SNP-heritability divided by the proportion of SNPs in each chromosome. The height of the bar represents the point estimate of the enrichment of heritability, and the error bars indicate the standard error of this point estimate.

We performed pooled as well as sex-specific X chromosome-wide association study (XWAS) meta-analyses using EU-RLS-GENE and 23andMe data (Methods). Based on the pooled meta-analysis, SNP-based heritability ${h}_{{\rm{pooled}}}^{2}$ carried by the X chromosome was 0.0035 (s.e. = 0.0010), with the sex-specific values again being lower in men (${h}_{{\rm{males}}}^{2}=0.0032$, s.e. = 0.0018) than in women (${h}_{{\rm{females}}}^{2}=0.0047$, s.e. = 0.0012; Extended Data Fig. 5 and Supplementary Table 5), but this difference was not significant (*P* = 0.49). Genetic correlation between the two sexes was high (*r*_g_ = 0.926, s.e. = 0.071, *P*_difference_ = 0.29, one-sample two-sided *Z*-test). Our analyses identified three independent risk loci for RLS on the X chromosome in the pooled data and one in the male-only data (Supplementary Tables 1 and 3).

### Replication of lead variants in additional datasets

**Extended Data Fig. 6:**
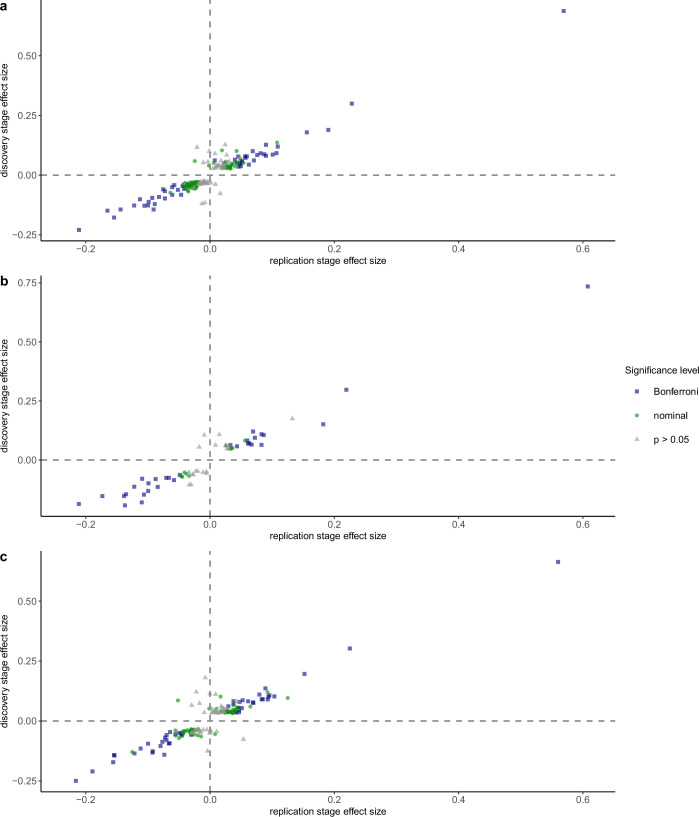
Replication of lead SNPs in independent validation samples. Association results of replication stage. The effect size (beta) of the replication analysis is plotted against the effect size (beta) of the discovery stage for genome-wide significant lead SNPs. The color-coding and symbol shape indicate the strength of the association signal in the replication stage meta-analysis (nominal two-sided P value of random-effects meta-analysis). Blue square, Bonferroni-corrected significance; green circle, nominal significance; grey triangle, not significant. **a**, Pooled meta-analysis with Bonferroni threshold set at 0.000255, correcting for 196 lead SNPs. **b**, Male-specific meta-analysis with Bonferroni threshold set at 0.00082, correcting for 61 lead SNPs. **c**, Female-specific meta-analysis with Bonferroni threshold set at 0.000318, correcting for 157 lead SNPs.

We combined data from three additional cohorts to replicate the lead SNP associations of our meta-analyses (Methods): the discovery dataset of a previously published meta-analysis, a second research participant sample from 23andMe and a second set of blood donors from INTERVAL, totaling 29,028 cases and 398,815 controls. Despite the considerably smaller sample size, 71% of the lead SNPs from the pooled discovery meta-analysis were at least nominally significant in the replication dataset (*P* < 0.05) and there was a high positive correlation between the effect size estimates of the discovery stage and the replication dataset (Pearson’s *r* = 0.94, *P* < 2.2 × 10^−16^; Extended Data Fig. 6a). The male- and female-specific analyses showed similar results (male, 67% of lead SNPs with *P* < 0.05, Pearson’s *r* = 0.97, *P* < 2.2 × 10^−16^; female, 70%, Pearson’s *r* = 0.92, *P* < 2.2 × 10^−16^; Extended Data Fig. 6b,c). A joint analysis of discovery and replication datasets revealed that all lead SNPs of the pooled, male-specific and female-specific meta-analyses reached Bonferroni-corrected significance (Supplementary Table 6).

### Functional annotation and biological interpretation

**Fig. 1:**
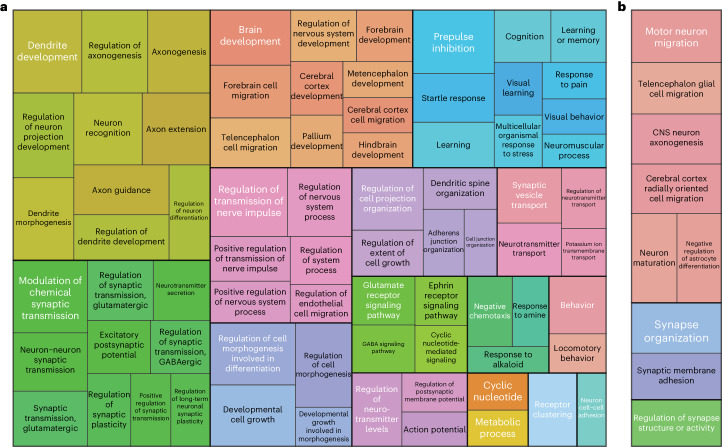
Pathway enrichment analysis. **a**,**b**, Treemaps of significantly enriched (FDR < 0.05, one-sample one-sided *Z*-test (DEPICT) or one-sided *t*-test (MAGMA)) pathways. Respective GO terms were clustered based on their semantic similarity (method: Wang, GOSemSim as implemented in the rrvgo package version 1.2.0) using results from DEPICT (**a**) and results from MAGMA (**b**). Terms are presented in rectangles. Coloring indicates the membership of a term in a specific cluster. In addition, each cluster is visualized by thick border lines. The size of each rectangle corresponds to the significance of the enrichment. The most significantly enriched term in each cluster was selected as the representative term and is displayed in white font.

We performed gene set and cell type enrichment analyses based on the pooled meta-analysis (Methods). We used DEPICT to perform gene set enrichment analyses across the genome-wide significant risk loci and detected 319 gene sets with a false discovery rate (FDR) < 0.05 (Supplementary Table 7). These clustered in pathways, processes and structures related to neurodevelopment, neuron migration, axon guidance, synapse formation and signal transduction between neurons (Fig. 1a). An additional gene set enrichment analysis using MAGMA prioritized nine biological processes related to neuron migration and synapse formation with an FDR <0.05 (Supplementary Table 8). This supported the results from DEPICT and emphasizes the key role of neurodevelopmental processes in RLS biology (Fig. 1b).

**Fig. 2:**
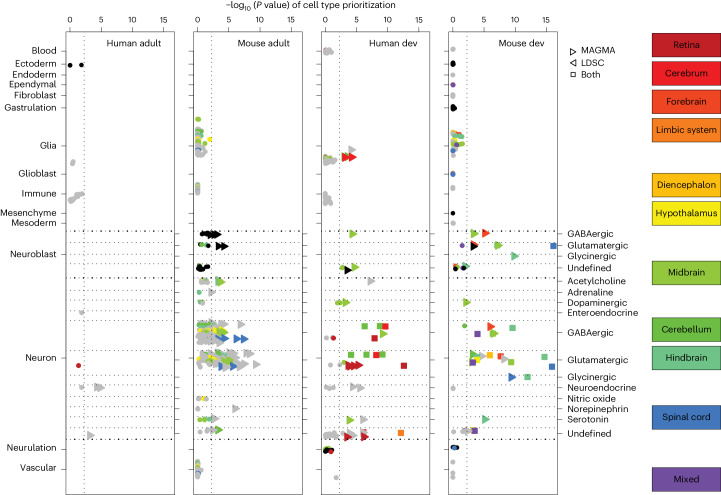
Tissue and cell type enrichment analysis. Cell type enrichment analysis results of mouse and human developmental (dev) and adolescent and adult CNS single-cell datasets. Cell types are annotated based on the class and subclass definitions used by the mouse brain atlases. We further annotated the neurons with their respective neurotransmitter type. Significance values (−log_10_ (*P* value) and FDR, one-sample one-sided *Z*-test) are reported for MAGMA-based methods, using MAGMA_Celltyping for human adult data and CELLECT-MAGMA otherwise. Gray and black dots indicate cell types not significant on the FDR level by both tools in any of the datasets; color alternates to separate neighboring cell types in the plot more clearly. The color code relates to the brain region where cells originated. Mixed refers to three cell types from the developmental-stage data, for which spatial mapping failed, resulting in an assignment to a mixture of brain regions (forebrain, midbrain, hindbrain) in varying proportions.

We performed enrichment analyses to identify tissue and cell types involved in RLS. We first examined body-wide human gene expression data. The default analysis in DEPICT identified 24 of 209 tissue and cell types with significant enrichment (FDR < 0.05), 23 of which were central nervous system (CNS) tissues (Supplementary Table 9). Using GTEx version 8 as an independent validation dataset yielded highly comparable results (Supplementary Table 10). Therefore, we focused on higher-resolution single-cell sequencing datasets of the nervous system in mice, available for developmental and postnatal stages (Methods). Only neurons and neuroblasts showed statistically significant enrichment, while glial and endothelial cells, for instance, did not (Fig. 2 and Supplementary Tables 11–14). We then dissected these cell types to identify specific anatomical regions and neurotransmitter classes (Fig. 2). We found cell types with statistically significant enrichment in all main compartments of the embryonic CNS: forebrain, midbrain, hindbrain and spinal cord. This was mirrored in the adult dataset, where cell types in the cerebrum, the cerebellum and the brainstem were highlighted. In most regions, both excitatory and inhibitory neuron types showed statistically significant enrichment, with glutamatergic neurons in the spinal cord showing the strongest enrichment. Overall, developmental-stage data yielded more robust enrichment than adult-stage data. Analyses in human datasets confirmed the enrichment in neuronal cell types and the higher level of significance obtained in the developmental datasets (Fig. 2 and Supplementary Tables 15 and 16). Again, excitatory and inhibitory neurons showed the highest enrichment. An additional analysis of bulk human brain transcriptome data from BrainSpan indicated an enrichment in the prenatal stage, but not the postnatal stage, underscoring a role for neurodevelopment in susceptibility to RLS (Supplementary Table 17).

We used diverse functional genomic annotation and fine-mapping approaches to build a sum score for ranking candidate causal genes within risk loci (maximum score = 12, Methods). Six loci contained no gene with a score above 2, 69 loci contained genes reaching a score of up to 6, and 89 loci contained genes with a score ≥ 7 (Supplementary Table 18). We focused further interpretation on the latter group. At 61 loci, there was a single independent lead SNP as well as a single top-scoring gene. These included six known loci, strengthening previous reports (*MEIS1*, *PTPRD*, *SKOR1*, *NTNG1*, *CADM1* and *RANBP17*)^3,4,10–13^. Because drug repurposing is one of the fastest options for translating GWAS findings into patient care, we mapped the top-scoring genes against the druggable genome and identified 13 potential candidates targeted by existing compounds (Table 1). Among them, *GRIA1* and *GRIA4*, which encode subunits of AMPA-type glutamate ionotropic receptors, provided genetic evidence of a link between RLS and glutamate receptor function. Another interesting candidate is *CCKBR*, which encodes the predominant cholecystokinin receptor in the brain^14,15^. Our prioritization algorithm also listed *SLC40A1*, which had already been identified in the discovery stage of a previous study but had failed to replicate^4^. *SLC40A1* encodes ferroportin 1, the only known transporter for iron export from cells, being relevant for iron replacement therapies^16–18^. To evaluate whether iron-related traits and RLS shared causal variants in *SLC40A1*, we performed additional colocalization analyses using recently published GWAS of peripheral iron measures as well as quantitative susceptibility mapping (QSM) and T2* magnetic resonance imaging data as readouts for brain iron levels^19–21^ (Supplementary Note). For the pallidum and the putamen, colocalization analysis pointed toward distinct causal variants (posterior probability for H_3_ hypothesis of coloc absolute Bayes factor analysis (PP.H3.abf)_pallidum_ ≥ 96.1% for QSM and PP.H3.abf_putamen_ > 99% for T2*), whereas results were inconclusive for the caudate nucleus. In other subcortical brain regions, the results were not statistically significant. For peripheral iron measurements, we saw a probability of >99% for different causal variants for both ferritin and total iron binding capacity and RLS. In general, our analyses suggest that the RLS association in the S*LC40A1* locus is distinct from iron-related associations (Supplementary Table 19).

**Table 1:** Drug repurposing options for top-scoring genes

GWAS locus lead SNP			Prioritized gene (score)	DrugBank-listed drugs or compounds	Druggability tier
ID	Position	*P* value			
rs10895816	11:105,285,122	1.16 × 10^−25^	*GRIA4* (10)	Talampanel, glutamic acid, CX-717	1
rs10839553	11:6,350,791	7.65 × 10^−16^	*CCKBR* (7)	Pentagastrin, cholecystokinin	
rs10038916	5:153,098,094	9.99 × 10^−16^	*GRIA1* (9)	Perampanel, lamotrigine, talampanel, glutamic acid, CX-717, CX516, tianeptine	
rs306960	8:142,005,245	1.18 × 10^−13^	*PTK2* (9)	Fostamatinib, endostatin	
rs12693542	2:190,445,848	1.35 × 10^−13^	*SLC40A1* (9)	Ferrous sulfate, tetraferric tricitrate decahydrate	
rs824920	2:222,786,280	1.35 × 10^−12^	*EPHA4* (7)	Fostamatinib	
rs714522	23:24,686,539	2.26 × 10^−8^	*POLA1* (7)	Fludarabine, clofarabine, cladribine, nelarabine	
rs2067133	5:102,364,542	7.59 × 10^−19^	*PAM* (11)	Copper, vitamin C	2
rs17123518	20:31,248,265	1.88 × 10^−11^	*DNMT3B* (7)	Decitabine	
rs56350804	2:217,560	3.55 × 10^−11^	*ACP1* (10)	Adenine	
rs72718216	14:69,455,773	3.23 × 10^−34^	*ACTN1* (9)	Copper, human calcitonin	3
rs11142701	9:73,762,953	6.01 × 10^−15^	*TRPM3* (11)	Primidone	
rs326779	11:29,617,859	3.81 × 10^−11^	*KCNA4* (7)	Dalfampridine	

Genes are named according to Ensembl gene name nomenclature and are mapped to the druggability tiers as provided by Finan et al.^33^. For each gene, the corresponding risk locus is indicated with its respective lead SNP and the corresponding two-sided *P* value from the pooled N-weighted genome-wide association meta-analysis (N-GWAMA). Approved and investigational drugs and small compounds targeting the products of these genes were extracted from the DrugBank Online database (release 5.1.8, https://go.drugbank.com/). ID, dbSNP rsID; position, chromosome:position on GRCh37 (hg19); score, sum score of gene prioritization in risk loci.

### Genetic correlation and MR analysis

**Fig. 3:**
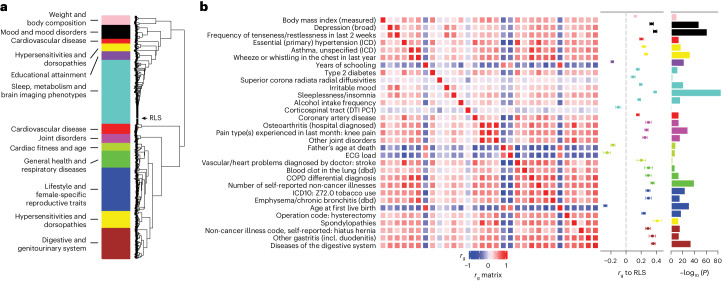
Genetic correlation analysis. **a**, Hierarchical clustering by a weighted correlation matrix analysis with the WGCNA package identified 11 modules. These were named with umbrella terms reflecting the type of traits contained in the respective module. **b**, Between-trait correlation matrix and genetic correlation with RLS for traits reflecting submodules identified in the 11 modules by consensus manual inspection of clusters. These traits were taken forward to MR analysis. The correlation matrix (*r*_g_ matrix) indicates genetic correlation between the individual traits. Genetic correlation of each trait with RLS is indicated in the second column (*r*_g_ to RLS); the significance of this correlation is reported in the third column (−log_10_ (*P* value), one-sample two-sided *Z*-test). COPD, chronic obstructive pulmonary disease; DTI PC1, diffusion tensor imaging principal component 1; ECG, electrocardiogram; dbd, diagnosed by doctor; ICD, ICD-10 coded hospital inpatient diagnosis; incl., including.

We performed a large-scale genetic correlation analysis followed by MR to discover potentially modifiable risk factors for RLS and to explore epidemiological or mechanistic overlaps with other diseases (Methods). Calculating genetic correlations with LDSC identified 1,054 of 2,649 analyzed traits and diseases as significantly correlated with RLS (FDR < 0.05; Supplementary Table 20). To factor in the complex interrelations between these traits, we performed bi-serial genetic correlation followed by weighted correlation network analysis of all 1,054 traits. This clustering yielded 11 modules, which reflected independent higher-level trait categories linked to RLS (Methods and Fig. 3a). The genetic correlation results strongly converged on RLS being associated with lower general physical as well as mental health. They confirmed epidemiological associations with increased body weight, depression, hypertension, cardiovascular disease, diabetes and sleep disturbances (Fig. 3b). However, they also provided evidence for less well-described associations of RLS with lower educational attainment, higher risk of asthma and diseases of the digestive system. In line with the increased prevalence in females, we identified a cluster of female-specific traits such as age of first childbirth, hysterectomy, oophorectomy and excessive menstruation (blue module, Fig. 3a,b and Supplementary Table 20).

**Fig. 4:**
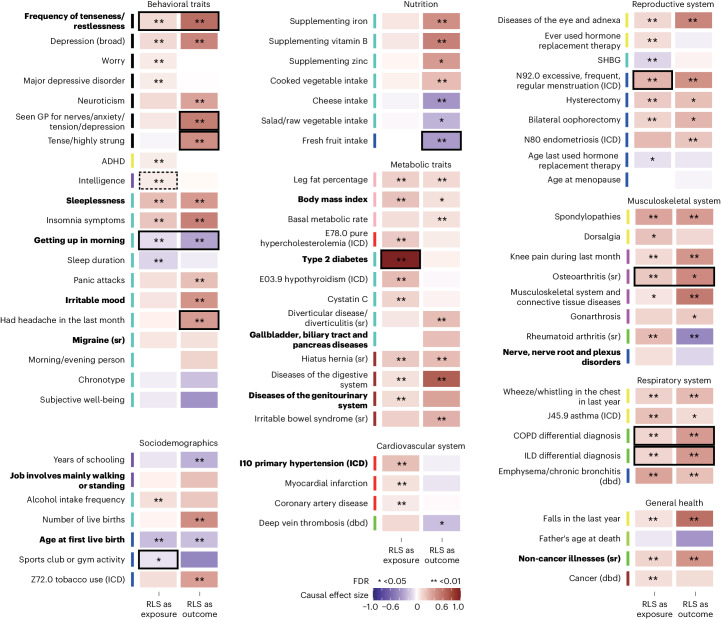
MR analysis. LHC-MR results for bidirectional MR between RLS and selected traits. The causal effect size is color coded, with dark blue indicating strong negative effects and dark red indicating strong positive effects. Significance of LHC-MR is reported as the FDR (LRT). Black borders mark traits for which LHC-MR analysis provided evidence for causal-only effects contributing to the relationship between the traits (*P*_LRT_causal_only_ < 0.05). Dashed black borders mark traits with evidence for confounding effects only in LHC-MR (*P*_LRT_latent_only_ < 0.05). Bold text indicates traits that showed consistent results in the IVW-MR analysis (one-sample two-sided *Z*-test, *P*_FDR_filter_ < 0.05 for significant effects and <0.05 for nonsignificant effects; Supplementary Table 22). ADHD, attention-deficit–hyperactivity disorder; GP, general practitioner; ILD, interstitial lung disease; SHBG, sex hormone binding globulin; sr, self-reported; *P*_LRT_, *P* value of the LRT from LHC-MR.

We performed MR to infer potential causal relationships between RLS and representative traits from these clusters (Fig. 4 and Supplementary Table 21). RLS as a common and complex disease is characterized by phenotypic heterogeneity and likely entails genetic pleiotropy, necessitating cautious interpretation of MR results. Therefore, we used the latent heritable confounder MR (LHC-MR) approach for the primary analysis, which is a robust method designed to account for pleiotropy and potential confounding (Methods). We confirmed known unidirectional and bidirectional relations, for example, that the number of live births significantly increased the risk of RLS or that insomnia symptoms and RLS were bidirectionally linked^8,9,22,23^.

For other traits, LHC-MR indicated relationships being causal rather than due to confounding. In terms of unidirectional relationships, RLS showed a significant effect (defined as *P*_FDR_ < 0.05) on type 2 diabetes with an effect estimate of *a*_RLS→diabetes2_ = 0.99 (s.e. = 0.06, *P*_FDR_ = 1.5 × 10^−68^) and significant likelihood-ratio tests (LRTs) for effects being only causal (*P*_LRT_causal_only_ = 8.5 × 10^−28^) and effects only of RLS on type 2 diabetes (*P*_LRT_only_RLS→diabetes2_ = 2.9 × 10^−40^). Unidirectional causal links to RLS with strong evidence were fresh fruit intake (decreased RLS risk with *a*_fruit→RLS_ = −0.33 ± 0.08, *P*_FDR_ = 0.0002, *P*_LRT_causal_only_ = 2.2 × 10^−5^, *P*_LRT_only_fruit→RLS_ = 2.3 × 10^−5^) and being tense or highly strung as well as having had a headache in the last month (elevated RLS risk with *a*_tense→RLS_ = 0.44 ± 0.06, *P*_FDR_ = 8 × 10^−12^, *P*_LRT_causal_only_ = 8.6 × 10^−9^, *P*_LRT_only_tense→RLS_ = 4.2 × 10^−8^ and *a*_headache→RLS_ = 0.37 ± 0.08, *P*_FDR_ = 2.9 × 10^−5^, *P*_LRT_causal_only_ = 1.2 × 10^−8^, *P*_LRT_only_headache→RLS_ = 6.9 × 10^−7^). Significant bidirectional relations with evidence of only causal effects were found for five traits (all with *P*_LRT_causal_only_ < 0.05; Fig. 4 and Supplementary Table 21): ease of getting up in the morning lowered RLS risk (*a*_ease→RLS_ = −0.3 ± 0.06) and vice versa (*a*_RLS→ease_ = −0.09 ± 0.02). The frequency of tenseness or restlessness in the last 2 weeks as well as two traits reflecting lung function increased RLS risk and vice versa, with a stronger effect on RLS (*a*_tenseness→RLS_ = 0.62 ± 0.07, *a*_RLS→tenseness_ = 0.11 ± 0.02, *a*_COPD-differential-diagnosis →RLS_ = 0.38 ± 0.06, *a*_RLS→COPD-differential-diagnosis_ = 0.12 ± 0.03), while, for self-reported osteoarthritis, RLS had the stronger effect (*a*_osteoarthritis→RLS_ = 0.46 ± 0.19, *a*_RLS→osteoarthritis_ = 0.18 ± 0.04). We performed inverse-variance weighted (IVW)-MR analyses with Steiger filtering and MR-Egger intercept assessment as a secondary analysis. The results were consistent for 14 traits, which included the unidirectional link between RLS and type 2 diabetes (Fig. 4 and Supplementary Table 22).

Considering the proposed involvement of brain iron homeostasis in RLS^24^ and *SLC40A1* as a candidate gene in our GWAMA, we also investigated peripheral and brain iron traits. Both genetic correlation and MR analyses did not reveal strong effects (Supplementary Tables 21 and 23). Only white matter hyperintensity measured by T2* was significantly correlated with RLS in the full dataset (*r*_g_ = 0.126, s.e. = 0.046, *P* = 0.0065, *P*_FDR_ = 0.016, one-sample two-sided *Z*-test). LHC-MR revealed a significant effect of peripheral calculated transferrin levels on RLS; however, this appears to be largely attributable to confounding factors (*P*_LRT_latent_only_ = 0.005).

### Development and validation of a risk prediction model

We assessed the predictive performance of basic linear models as well as that of models integrating interaction effects and time-dependent effects using genetic data and basic demographic variables such as age, sex and age of disease onset (Methods and Supplementary Note). We employed three classes of models, generalized linear models (GLMs) with or without interaction terms, random forest (RF) models and deep neural network (DNN) models, implemented as a binary or a time-to-event (survival) classifier. Genetic risk was calculated as a polygenic risk score (PRS) using individual dosages of 216 genome-wide significant SNPs (PRS.lead), because this score showed better performance than a score using genome-wide data (LDpred2) with an area under the receiver operator characteristic curve (AUC) of AUC_LDpred2_ = 0.66 ± 0.019 compared to AUC_PRS.lead_ = 0.73 ± 0.018 (*P* = 0.0056, two-sample two-sided *Z*-test).

**Fig. 5:**
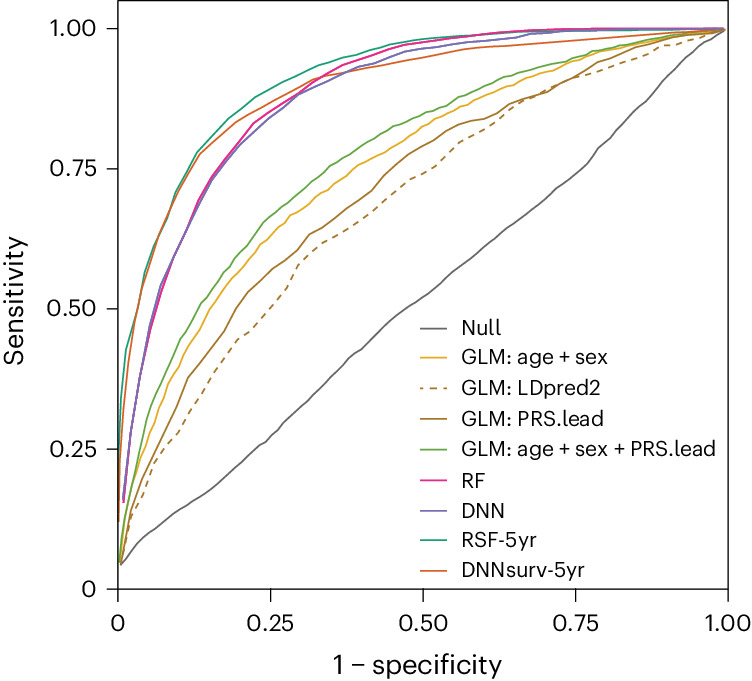
Risk prediction. Receiver operator characteristic curve showing the performance of different models used to predict RLS risk in the synthetic population representing the EU-RLS-GENE cohort. Null refers to the model including only the intercept (*y* ≈ 1). GLM:age + sex refers to the model including age, sex and principal components (PCs). GLM:LDpred2 refers to the model including the genome-wide PRS calculated with LDpred2-auto. GLM:PRS.lead refers to the model including the PRS based on 216 lead SNPs. GLM:PRS.lead + age + sex refers to the model including age, sex and the PRS based on 216 lead SNPs. RF refers to the RF model. DNN refers to the DNN model. RSF-5yr refers to the RF survival analysis for a 5-year period. DNNsurv-5yr refers to the DNN survival analysis for a 5-year period.

Overall, the machine learning survival classifier models considering nonlinear interactions and time-varying effects performed best (Fig. 5). The random survival forest model (RSF-5yr; 5-year period) and the DNN survival model (DNNsurv-5yr) showed comparable performance: AUC_RSF-5yr_ = 0.91 ± 0.008 compared to AUC_DNNsurv-5yr_ = 0.90 ± 0.012 in the EU-RLS-GENE dataset and AUC_RSF-5yr_ = 0.87 ± 0.005 compared to AUC_DNNsurv-5yr_ = 0.86 ± 0.012 in the INTERVAL dataset. Additional performance metrics such as odds ratio (OR) and area under the precision–recall curve yielded the same trends (Supplementary Table 24).

We also evaluated the contribution of the interaction effects to the model performance either directly (GLMs) or indirectly by calculating the incremental gain in explained variance for the DNN and RF models (Nagelkerke’s pseudo-*R*^2^; Methods). For the GLM, we found a significant interaction between PRS and age (*β* = −0.47, s.e. = 0.08, *P* = 4.3 × 10^−9^, one-sample two-sided *Z*-test). The impact of the PRS was significantly lower in the 60+ age group (OR_overall_ = 5.05 (4.69–5.45), OR_60+_ = 3.70 (3.27–4.19), *P*_difference_ = 2.6 × 10^−5^, two-sample two-sided *Z*-test). We did not find a significant sex difference in overall PRS effects (OR_male_ = 4.70 (4.16–5.31), OR_female_ = 5.28 (4.80–5.80), *P*_difference_ = 0.141, two-sample two-sided *Z*-test), even though the effect of sex was highly significant (OR = 2.54 (2.33–2.78), *P* = 1.93 × 10^−94^, one-sample two-sided *Z*-test). In the best-performing RF and DNN binary classification models, pseudo-*R*^2^ was 0.329 (s.e. = 0.003) and 0.324 (s.e. = 0.005), almost 1.5 times higher than in the GLM (*R*^2^ = 0.221, s.e. = 0.003). The time-to-event classifier models showed a further increase in *R*^2^ by approximately 10% for both models (${{R}}_{{\rm{RSF}}{\hbox{-}}5{\rm{yr}}}^{2}=0.363$, s.e. = 0.004; ${{R}}_{{\rm{DNNsurv}}{\hbox{-}}5{\rm{yr}}}^{2}=0.354$, s.e. = 0.005). Overall, nonlinear relationships and interactions accounted for 39.1% (s.e. = 1.96%) of the explained variance.

## Discussion

Performing the largest meta-analysis of RLS GWAS to date, we have increased the number of known risk loci eightfold. We included three cohorts, representative of commonly used strategies to assess behavioral phenotypes, ranging from in-person interviews to a single online question. They also reflect the breadth of target populations for recruitment into GWAS, including clinical cohorts as well as samples from the general population. Despite this heterogeneity, genetic correlations were strong between the cohorts, justifying their combination in a multivariate meta-analysis.

We investigated sex-specific genetic susceptibility in RLS. While the heritability was significantly higher in women, the genetic correlation between the sexes was close to one. Results from our simulation study pointed to an unobserved environmental risk factor and corresponding gene–environment interactions driving the difference in heritability. Our analyses emphasize the importance of tracking environmental exposures in genetically susceptible individuals and may motivate re-interpretation of previous observations in RLS, for example, of parity potentially driving the higher prevalence observed in women^8,9^. In line with the high genetic correlation between the sexes, there were only six loci where risk variants showed significant sex differences in effect size. An additional two loci in males and 21 loci in females were genome-wide significant in only one sex but did not reach significance in the between-sex heterogeneity tests. With larger sample sizes, some of these may turn out to be true sex-specific association signals.

Our enrichment analyses corroborate results from earlier GWAS of RLS by prioritizing CNS tissues and primarily pathways linked to neurodevelopment and neurotransmission^3^. Interestingly, the enrichment effects were consistently stronger in fetal and prenatal datasets. This suggests that development may represent a critical period in which genetic contributors to RLS susceptibility act on the activity, connectivity or composition of neurons in the CNS. Analyses in developmental mouse CNS single-cell data prioritized excitatory glutamatergic neurons in the spinal cord, hindbrain, midbrain and forebrain but also γ-aminobutyric acid (GABA)ergic neurons in at least the midbrain and hindbrain. This diversity was reflected in the adult dataset, with again mostly excitatory neurons showing enrichment. Overall, the diversity of cell types and structures with significant enrichment corresponds to the complex phenotype of RLS, which includes sensory and motor symptoms as well as a circadian pattern. Unfortunately, the current scarcity of high-resolution data limits the ability of our study to validate these observations in humans. Tissue enrichment analysis depends on the methodology as well as on the composition of the datasets. Specifically, definite exclusion of cell types is difficult as they may not have been represented in the dataset. We tried to address these limitations by using two different enrichment methods as well as several datasets.

Interestingly, except for the prioritization of *SLC40A1* (ferroportin), we did not identify strong links between iron metabolism and genetic risk factors for RLS in our pathway and genetic correlation analyses. However, the T2* and QSM values we used as surrogates for brain iron content are differentially influenced by iron and myelin; therefore, future magnetic resonance imaging GWAS with higher anatomical resolution may allow better dissection of genetic effects involved in iron and myelin content^25,26^. Moreover, we cannot rule out an incomplete representation of brain or general iron homeostasis in the currently available pathway definitions.

Our study provides discoveries relevant for advancing clinical care in RLS. We identified several genes that are druggable and in some cases targets of known drugs. For example, the prioritization of two glutamate receptors suggests that the efficacy of anticonvulsants in RLS should be re-assessed. Small open trials have shown good response to glutamate receptor antagonists such as perampanel or lamotrigine in RLS^27,28^. The benefit of α_2_δ ligands such as pregabalin or gabapentin adds further evidence that anti-epileptic drugs could be an additional therapeutic option^29^. Investigation into a completely new line of treatment is suggested by the prioritization of the cholecystokinin B receptor, a neuropeptide receptor that has been linked to pain modulation and anxiety-related behavior^15,30^. Furthermore, our genetic correlation and MR analyses identified relationships of potential medical relevance between RLS and several traits. In line with previous reports, the strongest genetic correlations with RLS were observed for insomnia symptoms and for depression^22,23^. MR analysis showed bidirectional effects, with the full model (causal as well as confounding effects) performing best. Probably, both pleiotropic genetic effects as well as the presence of RLS cases in the depression and insomnia cases and vice versa are involved. Disentangling the contributions of shared genetics and of case misclassification to this relationship will require large datasets with high-quality phenotyping of both insomnia and RLS. We saw a robust and significant unidirectional relationship of RLS with type 2 diabetes, with consistent results between LHC-MR and standard IVW-MR. The causal-only-effect model performed best in LHC-MR, suggesting that this link from RLS to diabetes is unlikely due to a heritable confounder. Thus far, cross-sectional and clinical studies have yielded inconsistent results regarding the causal relationship between RLS and type 2 diabetes^31^. Our MR analyses support a causal effect of RLS increasing the risk of type 2 diabetes. We found likely causal, albeit bidirectional relationships between RLS and osteoarthritis and between RLS and diseases of the respiratory system. Clinical or epidemiological studies on RLS in these disorders are limited or even non-existent at present; therefore, patients could benefit from increased awareness and research activities. The beneficial effect of modifiable behaviors on reducing the risk of RLS is underscored by findings that a healthy lifestyle, for example, fresh fruit consumption, is linked to lower RLS risk. Due to the inherent limitations of MR analysis, these results should not be overinterpreted. Even though the LHC-MR approach seems robust across a range of scenarios with different violations of the MR assumptions, it has its own drawbacks^32^. Therefore, we advise leveraging our findings to inform future clinical and epidemiological research aimed at gathering further evidence to support causality.

Predicting the likelihood of developing RLS is crucial for targeted disease-prevention strategies. We compared traditional PRSs to more advanced machine learning approaches integrating interaction and nonlinear effects. The latter showed superior performance compared to simple PRS-only or PRS-plus-linear interactions models. In our simulation study with only limited phenotypic data, the RF and DNN approaches provided comparable results. Enhanced phenotypic data may amplify the effectiveness of DNNs for predictive purposes. Two aspects limited our options for risk prediction. First, the definitive RLS cases (diagnosed by face-to-face interviews) with individual-level data required for developing the models had no detailed clinical data. Second, they were part of a case–control cohort and therefore do not reflect the general population structure, which necessitated creating a simulated dataset from the original data. Nevertheless, we were able to achieve an AUC of up to 91% for the 5-year prediction window with the machine learning approaches and validated our results in the INTERVAL study, where the performance was comparable with an AUC of up to 87%.

Collectively, our study marks a substantial advance in deciphering the genetic basis of RLS and paves the way for improving treatment and prevention strategies. We acknowledge two important limitations. First, biobank-scale longitudinal datasets with detailed medical and lifestyle information and high-quality RLS phenotyping are lacking. This type of data is needed to dissect the relationships discovered by genetic correlation and MR analyses as well as to study the roles of age, sex and other environmental effects and their interactions in shaping the risk and course of disease. Second, large-scale GWAS for RLS are currently limited to populations of European ancestry. An extension to non-European populations is imperative to improve genetic fine-mapping at shared loci and to adapt disease concepts to these populations with respect to non-shared genetics.

## Methods

### Ethics statement

All studies were approved by the respective local ethical committees, and all participants provided informed consent. The EU-RLS-GENE study was approved by an institutional review board at the University Hospital of the Technical University of Munich (2488/09). The INTERVAL dataset was approved by the National Research Ethics Service Committee East of England—Cambridge East (REC 11/EE/0538). Participants of 23andMe provided informed consent under a protocol approved by the external AAHRPP-accredited IRB, Ethical and Independent (E&I) Review Services. As of 2022, E&I Review Services is part of Salus IRB (https://www.versiticlinicaltrials.org/salusirb). The deCODE dataset was approved by the National Bioethics Committee of Iceland. The Danish Blood Donor Study (DBDS) dataset was approved by the Scientific Ethical Committee of Central Denmark (M-20090237) and by the Danish Data Protection agency (30-0444). GWAS studies in the DBDS were approved by the National Ethical Committee (NVK-1700407). The Emory dataset was approved by an institutional review board at Emory University, Atlanta, GA, USA (HIC ID 133-98).

### GWAS phenotyping and genotyping

Some of the samples were included already in our previous GWAS meta-analysis^3^. The reported sample numbers are the final sample numbers after quality control. Additional details are provided in the Supplementary Note.

#### Discovery meta-analysis

##### International EU-RLS-GENE consortium (7,248 cases (2,479 males and 4,769 females) and 19,802 controls (10,422 males and 9,380 females))

RLS cases were recruited in specialized outpatient clinics for movement disorders and in sleep clinics in European countries (Austria, Czech Republic, Estonia, Finland, France, Germany and Greece), Canada (Quebec) and the USA. RLS was diagnosed in a face-to-face interview by an expert neurologist or sleep specialist based on IRLSSG diagnostic criteria^1^. Controls were either population-based unscreened controls (Austria, Estonia, Finland, France, Germany) or healthy individuals recruited in hospitals (Canada, Czech Republic, Greece, USA). A total of 6,228 cases and 10,992 ancestry-matched controls had been genotyped on the Axiom array and were the study sample used in our previous meta-analysis. For the current study, 1,020 cases and 8,810 ancestry-matched controls were added who were genotyped on the Infinium Global Screening Array-24 version 1.0. Genotype calling was performed in GenomeStudio 2.0 according to the GenomeStudio Framework User Guide, and identical quality-control criteria were used for both datasets. Imputation was performed on the UK10K haplotype and 1000 Genomes Phase 3 reference panel using the EAGLE2 (version 2.0.5) and PBWT (version 3.1) imputation tools as implemented in the Sanger imputation server. Imputed SNPs with pHWE ≤ 1 × 10^−5^ or an INFO score < 0.5 were filtered out.

##### INTERVAL study (3,491 cases (1,291 males and 2,200 females) and 23,741 controls (12,511 males and 11,230 females))

The INTERVAL study includes whole-blood donors recruited in England between 2012 and 2014. The Cambridge-Hopkins Restless Legs questionnaire was used to define RLS cases, and probable and definite cases were combined to form a binary phenotype as described previously^3^. A detailed description of Axiom ‘Biobank’ array genotyping and the imputation procedure plus related quality control in the INTERVAL trial can be found elsewhere^34^. Briefly, imputation was performed using a joint UK10K and 1,000 Genomes Phase 3 (May 2013 release) reference panel via the Sanger imputation server, and variants with MAF ≥ 0.1% and INFO score ≥ 0.4 were retained for analysis.

##### Research participant cohort for 23andMe (105,908 cases (34,544 males and 71,364 females) and 1,502,923 controls (678,661 males and 824,262 females))

This study includes research participants of 23andMe who agreed to participate in research studies. The RLS phenotype was defined by self-reported responses to survey questions that assessed whether someone had ever been diagnosed with RLS or had ever received treatment for RLS as described previously^3^. Participants were genotyped on one of five platforms, all using Illumina arrays with added custom content (HumanHap550+ BeadChip, OmniExpress+ BeadChip, Infinium Global Screening Array). Participant genotype data were imputed in a two-step procedure using a reference panel created by combining the May 2015 release of the 1000 Genomes Phase 3 haplotypes with the UK10K imputation reference panel. Pre-phasing was carried out using either the internally developed tool Finch, which implements the Beagle algorithm, or EAGLE2. Imputation was performed with Minimac3.

#### Replication meta-analysis

##### Research participant cohort for 23andMe (19,214 cases and 347,000 controls)

This cohort includes only individuals who had not been part of the 23andMe GWAS used in the discovery meta-analysis. Cases and controls were defined as described above.

##### INTERVAL replication cohort (1,591 cases and 10,000 controls)

Individuals in this cohort do not overlap with samples included in the INTERVAL GWAS used in the discovery meta-analysis. RLS status was assessed with a single question on having received a diagnosis of RLS.

For 23andMe and INTERVAL, genotyping and imputation was carried out as described for the discovery stage.

##### deCODE–DBDS–Emory cohort (8,223 cases and 41,815 controls)

This dataset included the DBDS, a cohort from deCODE Genetics, Iceland, the Emory Hospital Atlanta, USA and the Donor InSight-III study. Phenotyping and genotyping procedures have been described in detail previously^4^.

### SNP-based association analysis

#### Discovery-stage GWAS of autosomes

##### EU-RLS-GENE GWAS

First, the Axiom- and the GSA-genotyped datasets were analyzed separately using SNPTEST version 2.5.4 with genotype dosages and assuming an additive model. Age, sex and the first ten PCs from the MDS analysis in PLINK were included as covariates. These summary statistics of the two datasets were then combined by fixed-effect inverse-variance meta-analysis (STERR scheme) using METAL (release 2011-03-25)^35^. One round of genomic control was performed in each dataset before meta-analysis.

##### INTERVAL GWAS

Assuming an additive genetic model, genotype dosages were analyzed in SAIGE (0.35.8.8) using a linear mixed model to account for cryptic relatedness and saddle point approximation to account for case–control imbalance^36^. Age, sex and the first ten PCs of ancestry were included as potential genomic confounders. The analysis was restricted to genetic variants with MAF ≥ 0.001, INFO ≥ 0.4 and a minor allele count of 10.

##### The 23andMe GWAS

Association analysis was conducted by logistic regression (LRT) assuming additive allelic effects and imputed dosages. Age, sex, genotyping platform and the first ten PCs were included as covariates.

In all individual GWAS, sex-specific analyses were performed using the same pipelines as those for the pooled analyses minus adjustment for sex as a covariate.

#### Discovery-stage meta-analysis for autosomes

We applied the same methods for both the pooled and the sex-specific GWAS. The three independent datasets were combined in a multivariate GWAS meta-analysis using the *N*-weighted-GWAMA R function (version 1.2.6)^37^. To assess the possibility of heterogeneity of SNP effects between the studies, Cochran’s *Q*-test was applied as described in METAL.

#### Discovery-stage meta-analysis for chromosome X

Data for the X chromosome were available in two of the discovery-stage datasets: EU-RLS-GENE and 23andMe.

##### EU-RLS-GENE XWAS

For the pooled association analysis, male genotypes were coded as 0/2 (assuming no dosage compensation in males). All other methods were identical to those of the autosomal analyses. In sex-stratified analyses, males were coded as 0/1 and females as 0/1/2.

##### The 23andMe XWAS

In both pooled and sex-stratified analyses, males were coded as 0/2 and females as 0/1/2.

Pooled and sex-specific meta-analyses were performed using the N-GWAMA R function as in the autosomal analysis. Because N-GWAMA operates with *Z* scores, the type of male allele coding did not affect the results.

#### Sex-specific meta-analysis association analysis

We performed sex-specific (male-only and female-only) meta-analyses of the corresponding GWAS using the N-GWAMA approach as described above. The results were used to estimate sex-specific heritability and genetic correlation between the sexes.

To detect sex-specific effects, we tested all independent (*r*^2^ < 0.2) genome-wide significant SNPs of the pooled and sex-specific meta-analyses for heterogeneity of effect sizes between the two sexes using Cochran’s *Q*-test (one-sided) and a Bonferroni-corrected significance threshold of *P*_adj_ ≤ 0.05/221.

#### Replication-stage association analysis

For 23andMe and INTERVAL, quality control and statistical analysis were performed as described for the discovery stage. Statistical analysis for the DBDS, deCODE–Emory and Donor Insight studies has been described previously^4^. Meta-analysis was performed using Han and Eskin’s random-effects model in METASOFT (RE2, METASOFT version 2.0.1)^38^.

#### Identification of risk loci and independent lead SNPs

To define independent risk loci, we first used the ‘--clump’ command in PLINK (version 1.90b6.7)^39^ to collapse multiple genome-wide significant association signals based on linkage disequilibrium (LD) and distance (clump-r2 > 0.05, clump-kb < 500 kb clump-p1 < 5 × 10^−8^, clump-p2p-value < 10^−5^). We then performed conditional analyses to identify secondary independent signals in risk loci using GCTA (version 1.93.0beta) with the ‘-cojo-slct’ option, the *P*-value threshold for genome-wide significance set at 5 × 10^−8^, the distance window set at 10 Mb and the colinearity cutoff set at 0.9 (ref. ^40^). LD was derived from EU-RLS-GENE genotype data. Independent genome-wide significant signals were merged into one genomic risk locus if either their LD block distance was <500 kb or their clumped regions were overlapping.

### Heritability analyses

Heritability is reported on the liability scale unless otherwise indicated. Prevalence estimates were derived from the population cohorts INTERVAL and 23andMe themselves. For the EU-RLS-GENE case–control dataset and for the meta-analysis, prevalence estimates were derived from previous publications on European ancestries.

We estimated SNP-based heritability under several different heritability models. LDSC (version 1.0.1) was used with standard settings, invoking a model where SNPs with different MAFs are expected to contribute equally to heritability^41^. LDAK (version 5.0) was used with standard settings to implement the LDAK model, where SNP contributions depend on LD structure and MAF as well as the BLD-LDAK and BLD-LDAK+Alpha models, which incorporate additional annotation-based features^42^. All analyses were based on summary statistics and filtering according to LDSC default settings, that is, HapMap3 non-HLA SNPs with MAF > 0.01 and INFO ≥ 0.9. The Akaike information criterion of each of these models was reported for model comparison. Further details are provided in the Supplementary Note.

For X chromosome heritability estimation, we followed the approach described by Lee et al. and used the summary statistics of the N-GWAMA meta-analysis^43^. For sex *k*, the SNP heritability ${h}_{k}^{2}$ relates to the expected *χ*^2^ statistics as ${\mathbb{E}}({\chi }_{k}^{2})\approx 1+{N}_{k}{h}_{k}^{2}/{M}_{{\rm{eff}}}$, where *N*_*k*_ is the GWAS sample size, and *M*_eff_ is the effective number of loci within the examined genomic region (assumed to be the same in males and females). For calculation of the (sex-specific) relative heritability contribution of the X chromosome, *χ*^2^ statistic-based *h*^2^ was also calculated for the autosomes.

### Genetic correlation analysis

For autosomal data, genetic correlations were calculated using LDSC (version 1.0.1) using the same SNP filtering criteria and the two-step estimation option as in the heritability estimation. Because the LDSC framework is not applicable for chromosome X, the genetic correlation coefficient ${\hat{r}}_{\rm{g}}$ was estimated as ${\hat{r}}_{\rm{g}}=\,\frac{\widehat{{Z}_{\rm{m}}{Z}_{\rm{f}}}}{\sqrt{(\;{\hat{\chi }}_{\rm{f}\,}^{2}-\,1)(\;{\hat{\chi }}_{\rm{m}\,}^{2}-\,1)}}$, where *Z* and *χ*^2^ are the *Z* scores and mean *χ*^2^ estimates from the female (f) and male (m)-specific studies.

In addition to between-study and between-sex genetic correlations, we performed a large-scale genetic correlation screen for RLS (represented by the pooled autosomal meta-analysis data) and other traits using LDSC as described above. Sources and filtering criteria for summary statistics included in this screen are provided in the Supplementary Note.

Traits significantly correlated with RLS (FDR < 0.05, one-sample two-sided *Z*-test) were taken forward to a bi-serial genetic correlation analysis. Here, we computed the pairwise ${\hat{r}}_{\rm{g}}$ between all traits.

An unsigned weighted correlation matrix was built using the pairwise ${\hat{r}}_{\rm{g}}$ and used as input for a weighted correlation matrix analysis to perform hierarchical clustering and to detect modules with the WGCNA package (version 1.69)^44^. The following settings were applied in WGCNA: softPower, 6; network type, ‘unsigned’; TOMDenom, ‘min’; Dynamic-cutree, method = ‘hybrid’; deepSplit, 2; minModuleSize, 30; pamStage, TRUE; pamRespectsDendro, FALSE; useMedoids, FALSE. The defining trait categories in each module were determined by consensus through independent review of the within-module cluster structure by visual inspection of network plots at two sites (Helmholtz and Cambridge).

### Mendelian randomization

To select traits for MR, we defined two to eight clusters in a module based on its complexity. In each cluster, the traits were ranked according to the significance of their correlation with RLS, and we selected the most significantly correlated medical conditions or potentially modifiable lifestyle factors. We supplemented this list with traits for which an association with RLS has been described in the literature.

Using R version 4.0.4, we filtered GWAS datasets to uncorrelated SNPs (*r*^2^ < 0.01 in the European 1000 Genomes Phase 3 data), aligned them to GRCh37 and mapped them to dbSNP 153 with the gwasvcf package (version 0.1.0). We harmonized effect alleles across studies using the TwoSampleMR package (version 0.5.6)^45^. Palindromic variants with ambiguous allele frequencies and those with unresolved strand issues were excluded from analysis.

To avoid violations of the classical MR assumptions when studying correlated and likely pleiotropic traits, we used a robust method for bidirectional MR, LHC-MR (version 0.0.0.9000)^32^. Traits with low heritability (*h*^2^ < 2.5%, ${P_{h^2}}$ > 0.05) were excluded from the analysis. Significance of directionality and confounding effect were tested by comparing the goodness of fit of six degenerate LHC-MR models (only latent effect, only causal effect, only causal effect to RLS, only causal effect from RLS, no causal effect to RLS and no causal effect from RLS) to the full model. We supplemented these analyses with those based on the IVW and MR-Egger methods.

### Gene prioritization in risk loci

All analyses were performed on the N-GWAMA results of the pooled meta-analysis. We applied several complementary approaches to prioritize candidate genes in the genome-wide significant risk loci. These included the gene-prioritization pipeline of DEPICT (version 1.rel194), three prioritization workflows (positional, eQTL-based and topology-based mapping) provided on the FUMA platform (https://fuma.ctglab.nl/, version 1.3.6a), a gene-level GWAS using MAGMA version 1.08, a transcriptome-wide association study using S-PrediXcan and S-MultiXcan (MetaXcan package version 0.7.4), a colocalization analysis with eCAVIAR (version 2.2) and statistical fine-mapping with CAVIARBF (version 0.2.1)^46–52^. In the DEPICT, FUMA eQTL-based mapping, MAGMA and transcriptome-wide association study analyses, a gene was considered prioritized if it had an FDR < 0.05; in FUMA topology-based mapping, if it had an FDR < 1 × 10^−5^; and in eCAVIAR, if it had a colocalization posterior probability > 0.1. In FUMA positional mapping, a gene was considered prioritized if genome-wide significant SNPs physically mapped to it. In statistical fine-mapping, a gene was considered prioritized if an SNP in the 95% credible set of the risk locus could be linked to it by either eQTL, chromatin interaction or positional mapping. In addition, we checked whether a gene contained genome-wide significant coding variants (the gene was considered prioritized if it did) and whether a gene mapped to a gene set that was significant in our enrichment analyses (the gene was considered prioritized if it did). We combined the results of all approaches per gene in a prioritization score by summing up the individual results, counting ‘not prioritized’ as 0 and ‘prioritized’ as 1. Further details are provided in the Supplementary Note.

### Enrichment analyses

#### Gene set and pathway enrichment analyses

##### DEPICT

We ran DEPICT to detect enrichment of gene sets across risk loci as well as to identify tissue and cell types where expression is enriched for genes across risk loci. We set the significance thresholds for lead SNPs at 1 × 10^−5^ and at 5 × 10^−4^ for null GWAS; all other settings were the same as those used for gene prioritization (see above). DEPICT was run with all built-in datasets. eQTL mapping and functional prioritization were evaluated in DEPICT’s built-in eQTL and reconstituted gene sets.

Excluding 12 SNPs not reaching genome-wide significance in the joint analysis of discovery and validation did not change the main results (Supplementary Table 25).

##### MAGMA

MAGMA (version 1.08) was used to perform gene set enrichment testing for pathway identification. MAGMA conducts competitive gene set tests with correction for gene size, variant density and LD structure. A total of 7,522 gene sets representing the GO biological process ontology (MSigDB version 7.1, C5 collection, GO:BP subset) were tested for association. We adopted a significance threshold of FDR < 0.05 (one-sided *t*-test).

#### Tissue and cell type enrichment analyses

Using the settings described above, we tested enrichment of RLS heritability with DEPICT across 209 different tissue types covered in the built-in dataset. For an independent validation on the tissue level as well as for the analyses on the cell type level, we mainly used the CELLEX and CELLECT tools^53^. CELLECT provides two different gene-prioritization approaches for heritability enrichment testing, S-LDSC and MAGMA covariate analysis^54,55^. For compatibility of the results, the summary statistics of the pooled N-GWAMA analysis were filtered using settings identical to those in our LDSC heritability analyses. Following the recommendations by Timshel et al.^53^, we applied a ‘tiered’ approach by starting with body-wide datasets and then focusing on CNS-centric datasets. We used CELLECT software (version 1.3.0) with default settings but updated to MAGMA version 1.08 to test enrichment of RLS heritability in cell type- or tissue-specific genes for datasets with publicly available RNA-seq data. These analyses require a measure of expression specificity for each gene in a cell or tissue type. We either used CELLEX (version 1.2.1) to compute expression specificity or relied on precomputed CELLEX expression specificity scores. Human adult datasets without publicly available raw RNA-seq data were analyzed using MAGMA_Celltyping (version 2.0.0) in top10 mode. The list of input datasets is provided in the Supplementary Note, and results of our evaluation of both approaches showing high correlation are presented in Supplementary Fig. 1 and Supplementary Table 26.

### Risk prediction

We applied three types of models for genetic risk evaluation and RLS risk prediction: GLM with and without interaction terms, RF models and DNN models. These were implemented as binary classifiers as well as time-to-event classifiers.

Training of the models and evaluation by tenfold cross-validation were based on the EU-RLS-GENE Axiom subset. Therefore, we first conducted a meta-analysis excluding this dataset to generate unbiased summary statistics to be used in all models. Because GWAS have an ascertainment bias, we constructed a simulation cohort dataset by resampling of the EU-RLS-GENE Axiom subset based on the year of birth of the sampled individuals, their ages at onset and the demographic composition of the German population (Supplementary Note). We calculated the PRS using dosages of 216 independent lead SNPs of our discovery meta-analyses.

For a baseline comparison of the predictive power of this score to a PRS based on genome-wide data, we calculated a genome-wide PRS using the LDpred2-auto option of LDpred2 (R package bigsnpr version 1.12.2)^56^. Variants and the LD reference panel were based on the HapMap3 EUR dataset, and window size for calculating SNP correlation was set to 3 cM.

Binary classification models were evaluated by Nagelkerke’s pseudo-*R*^2^, receiver operator characteristic AUC and precision–recall AUC. A 5-year binary classifier was constructed for each of the time-to-event models by predicting the label until the next 5 years and evaluated by the metrics for binary classification.

To evaluate the contribution of the interaction effects to model performance, we estimated the effect sizes of interaction terms such as PRS × age by logistic regression:

$$\begin{array}{l}P({\rm{RLS}}=1|{\rm{PRS}},{\rm{sex}},{\rm{age}},{\bf{PC}})\\=\displaystyle\frac{1}{1+{e}^{-\left({\beta }_{0}+{\beta }_{1}{\rm{PRS}}+{\beta }_{2}{\rm{sex}}+{{\beta }}_{3}{\rm{age}}+{\beta }_{4}{\rm{PRS}}\times {\rm{sex}}+{{\beta }}_{5}{ {\rm{PRS}\times\rm{age}}}+{{\beta }}_{6}{{\rm{sex}}\times\rm{age}} +{{\beta }}_{7}{{\rm{PRS}}\times {\rm{sex}\times\rm{age}}}+{\boldsymbol{\gamma }}\cdot{\bf{PC}}\right)}},\end{array}$$

where age is the dummy variable of age in bins of 20 years, **PC** indicates the first ten PCs from the MDS analysis in PLINK, **γ** is a vector of effect sizes of PCs and the PRS = Σ_*j*_*w*_*j*_*g*_*j*_, where *w*_*j*_ and *g*_*j*_ are the per-allele effect size and dosage of the *j*-th SNP, respectively.

For the DNN and RF models, we used these logistic regression estimates as the baseline and then further estimated the interaction effect sizes indirectly by calculating the incremental gain in explained variance (Nagelkerke’s pseudo-*R*^2^) from model_0_ to model_1_ as:

$${R}^{2}=\left(1-\left(L\left(\rm{model}_{0}\right)/{\it{L}}(\rm{model}_{1})\right)^{\frac{2}{\it{N}}}\right)\left(1-{\it{L}}(\rm{model}_{0})^{\frac{2}{\it{N}}}\right)^{-1},$$

where *L* is the likelihood function for a logistic regression model with the first ten PCs included as covariates.

Binary classification models, GLMs and RF and DNN models were built, optimized and trained by H2O AutoML (version 3.36.0.2) in R (version 4.0.2)^57^. Time-to-event models were implemented with randomForestSRC (version 3.0.1) in R (version 4.0.2) and PyTorch^58^ (pycox version 0.2.1 and PyTorch version 1.6.0). Cross-validation-based Nagelkerke’s pseudo-*R*^2^ was calculated in R version 4.0.2.

### Reporting summary

Further information on research design is available in the Nature Portfolio Reporting Summary linked to this article.

## Online content

Any methods, additional references, Nature Portfolio reporting summaries, source data, extended data, supplementary information, acknowledgements, peer review information; details of author contributions and competing interests; and statements of data and code availability are available at 10.1038/s41588-024-01763-1.

### Supplementary information

Supplementary Information Supplementary Note and Fig. 1.

Reporting Summary

Peer Review File

Supplementary Tables Supplementary Tables 1–26.

## Figures and Tables

**Extended Data Table 1:**
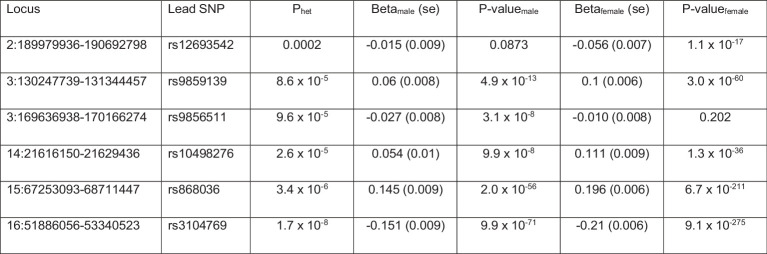
Lead SNPs with significant heterogeneity of effect sizes between sexes

Locus, chromosomal position of risk locus (chr:start-end, hg19); lead SNP, N-GWAMA lead SNP for locus from pooled meta-analysis; *P*_het_, nominal one-sided P value of Cochran’s Q test for heterogeneity; Beta_male_ and *P-value*_male_, effect size estimate and nominal two-sided P value of male-only meta-analysis; Beta_female_ and *P-value*_female_, effect size estimate and nominal two-sided P value of female-only meta-analysis.

## Data Availability

Summary statistics of the meta-analysis are publicly available for the top 10,000 SNPs at Zenodo (10.5281/zenodo.10804907)^59^. Summary statistics of the discovery-stage International EU-RLS-GENE consortium GWAS and the INTERVAL GWAS are available at the GWAS Catalog (https://www.ebi.ac.uk/gwas/) under accession codes GCST90399568, GCST90399569, GCST90399570, GCST90399571, GCST90399572 and GCST90399573. The full GWAS summary statistics for the 23andMe discovery dataset have been made available through 23andMe to qualified researchers under an agreement with 23andMe that protects the privacy of the 23andMe participants. Datasets have been made available at no cost for academic use. Please visit https://research.23andme.com/collaborate/#dataset-access/ for more information and to apply to access the data. Additional data used for tissue and cell type enrichment analysis are available here: developmental (http://mousebrain.org/development/downloads.html) and adult single-cell RNA-seq datasets (http://mousebrain.org/adult/downloads.html) from the Mouse Brain Atlas (http://mousebrain.org/), the Human Gene Expression During Development dataset from the BBI-Allen Single Cell atlases (https://descartes.brotmanbaty.org/), the BrainSpan Developmental Transcriptome RNA-seq dataset from the BrainSpan Atlas of the Developing Human Brain (https://www.brainspan.org/static/home), the V8 RNA-seq dataset (GTEx_Analysis_2017-06-05_v8_RNASeQCv1.1.9_gene_reads.gct.gz) from GTEx (https://gtexportal.org/home/datasets) and the human C8 collection from MSigDb version 7.4 (http://software.broadinstitute.org/gsea/msigdb/), with legacy versions available at https://www.gsea-msigdb.org/gsea/downloads_archive.jsp after creating a user account with GSEA–MSigDB. Summary statistics of GWAS for genetic correlation and MR analyses are available at the University of Bristol Integrative Epidemiology Unit OpenGWAS server (https://gwas.mrcieu.ac.uk) and the GWAS Atlas (https://atlas.ctglab.nl/). Additional GWAS summary statistics for iron-related traits are available at https://www.fmrib.ox.ac.uk/ukbiobank/gwas_resources/index.html, https://open.win.ox.ac.uk/ukbiobank/big40/BIGv2/ and https://www.decode.com/summarydata/. A complete list of sources used for annotation with FUMA is available at https://fuma.ctglab.nl/links and https://fuma.ctglab.nl/tutorial. Auxiliary files for use with MAGMA are available at https://ctg.cncr.nl/software/magma. Additional files for use with LDSC and LDAK are available at https://alkesgroup.broadinstitute.org/LDSCORE/. Information about drug targets is available at the free-to-access database DrugBank Online (https://go.drugbank.com/).

## Code availability

We provide information on publicly available software and settings in the Methods and the Supplementary Note. For custom data analysis, we describe the theoretical background as well as the models used in detail in the Supplementary Note. Custom code scripts are available on Zenodo (10.5281/zenodo.10804907)^59^. Publicly available software used in this study includes PLINK (version 1.90b6.7, https://www.cog-genomics.org/plink/1.9/), SNPTEST (version 2.5.4, https://www.chg.ox.ac.uk/~gav/snptest/), SAIGE (0.35.8.8, https://github.com/saigegit/SAIGE), N-GWAMA (version 1.2.6, https://github.com/baselmans/multivariate_GWAMA), METAL (release 2011-03-25, https://csg.sph.umich.edu/abecasis/metal/index.html), METASOFT (version 2.0.1, https://web.cs.ucla.edu/~eeskin/), GCTA (version 1.93.0beta, https://yanglab.westlake.edu.cn/software/gcta/#Overview), LDSC (version 1.0.1, https://github.com/bulik/ldsc), LDAK (version 5.0, https://dougspeed.com/ldak/), LHC-MR (version 0.0.0.9000, https://github.com/LizaDarrous/lhcMR), DEPICT (version 1 rel194, https://github.com/perslab/depict), FUMA (version 1.3.6a, https://fuma.ctglab.nl/), MAGMA (version 1.08, https://cncr.nl/research/magma/), MetaXcan (version 0.7.4, https://github.com/hakyimlab/MetaXcan), eCAVIAR (version 2.2, https://github.com/fhormoz/caviar), CAVIARBF (version 0.2.1, https://bitbucket.org/Wenan/caviarbf/src/master/), CELLECT (version 1.3.0) and CELLEX (version 1.2.1) (https://github.com/perslab/CELLECT), MAGMA_Celltyping (version 2.0.0, https://github.com/neurogenomics/MAGMA_Celltyping), pycox (version 0.2.1, https://github.com/havakv/pycox), PyTorch (version 1.6.0, https://github.com/pytorch/pytorch), H2O AutoML (version 3.36.0.2, https://docs.h2o.ai/h2o/latest-stable/h2o-docs/automl.html), the Sanger imputation server (https://imputation.sanger.ac.uk/), EAGLE2 (version 2.0.5, https://alkesgroup.broadinstitute.org/Eagle/), PBWT (version 3.1, https://github.com/richarddurbin/pbwt), Minimac3 (https://genome.sph.umich.edu/wiki/Minimac3), R (version 4.0.4 and version 4.0.2, https://cran.r-project.org/), the rrvgo package (version 1.2.0, https://bioconductor.org/packages/release/bioc/html/rrvgo.html), the WGCNA package (version 1.69, https://cran.r-project.org/web/packages/WGCNA/index.html), the TwoSampleMR package (version 0.5.6, https://mrcieu.github.io/TwoSampleMR/index.html), the coloc package (version 5.1.0, https://chr1swallace.github.io/coloc/), the BayesianTools package (version 0.0.10, https://github.com/florianhartig/BayesianTools), the bigsnpr package (version 1.12.2), implements LDpred2 (https://privefl.github.io/bigsnpr/), the gwasvcf package (version 0.1.0, https://github.com/MRCIEU/gwasvcf) and the randomForestSRC package (version 3.0.1, https://www.randomforestsrc.org/).
