# Supplementary material for: Genome-wide meta-analyses of restless legs syndrome yield insights into genetic architecture, disease biology and risk prediction
Source: Nat Genet. 2024 Jun 5;56(6):1090–9. doi: 10.1038/s41588-024-01763-1 (PMC11176086; doi:10.1038/s41588-024-01763-1)
Supplement: Supplementary file 1 — Supplementary Note and Fig. 1. [file 41588_2024_1763_MOESM1_ESM.pdf]

nature genetics

Supplementary information https://doi.org/10.1038/s41588-024-01763-1

Genome-wide meta-analyses of restless legs syndrome yield insights into genetic architecture, disease biology and risk prediction

In the format provided by the authors and unedited

Schormair, Zhao, Bell et al. Genome-wide meta-analyses of restless legs syndrome yield insights into genetic architecture, disease biology, and risk prediction.

Supplementary Information File

Contains:

Supplementary Note

Supplementary Figure 1

Consortium Membership Information

Supplementary Note

Discovery stage GWAS phenotyping

EU-RLS-GENE GWAS: RLS was diagnosed in a face-to-face interview by an expert neurologist or sleep specialist based on IRLSSG diagnostic criteria.[1]

RLS was diagnosed in a face-to-face interview by an expert neurologist or sleep specialist implementing the IRLSSG diagnostic criteria established by the IRLSSG in 2003. RLS cases had to fulfill all four diagnostic criteria evidence for secondary RLS had to be absent.

INTERVAL GWAS: The Cambridge-Hopkins Restless Legs Questionnaire was used to define RLS cases and probable and definite cases were combined to form a binary phenotype. This required participants to answer affirmatively to experiencing uncomfortable feelings/sensations in their legs when sitting or lying down accompanied by a need or urge to move their legs. These sensations needed to occur when participants were resting and typically improve upon movement. Furthermore, they needed not to be equally likely to occur throughout the day or most/least often in the morning/evening and night. Positional discomfort/leg cramp (common mimics of RLS) were also ruled out through the requirement that participants had to state that changing their leg position once was not sufficient to relieve these sensations and that they are not due to muscle cramps. The distinction between definite and probable cases was made based on whether these feelings were only present when sitting and lying/lying only (definite) or only when sitting (probable). Individuals who stated that they never had experienced uncomfortable feelings/sensations in their legs when sitting or lying down accompanied by a need or urge to move their legs were defined as controls.

23andMe GWAS: The RLS phenotype was defined by self-reported responses to survey questions which assessed whether someone has ever been diagnosed with RLS or has ever received treatment for RLS. Participants were classified as cases if they answered positively to any of these questions. Participants were classified as controls if they answered negatively. Participants who answered “I’m not sure” or “I don’t know” to any question were excluded.

Discovery stage GWAS genotyping quality control

EU-RLS-GENE GWAS: Individuals were removed if they showed a deviation ≥ 4 SD from the mean of the population MDS, thereby restricting to individuals of European ancestry, ambiguous sex calls, or an imiss-vs-het score ≥ 3 SD. For each pair of related individuals (defined as a PIHAT ≥ 0.09375), the sample with lower genotyping quality was removed. SNPs were excluded if they had a call rate < 98%, a MAF < 0.01, showed > 1 discordant genotype in duplicate samples, had a p-value for deviation from Hardy-Weinberg-Equilibrium (pHWE) ≤ 1 x 10-5 in controls, or were ambiguous.

INTERVAL GWAS: Before variant QC, duplicates and non-European ancestry samples were removed using autosomal variants with MAF > 0.05, HWE p-value > $1x10^{-6}$, and r2 ≤ 0.2. PLINK's Method-of-Moments IBD approach (PIHAT ≥ 0.9) and PCA (PC1 or PC2 scores < 0) identified duplicates and non-European ancestries, respectively. Variants were excluded for HWE deviation ($p < 5x10^{-6}$), low call rates (< 0.97), or if failing in four or more batches (out of ten). Sample contamination, assessed by the Jun et al. method[2], led to excluding samples with >10% contamination or 3%-10% contamination with >10 close relatives. Additional exclusions were made for heterozygosity outliers and samples with phenotypic sex data issues. For IBD and PCA, ~100,000 high-quality were selected and LD-pruned (r2 < 0.1) to form an uncorrelated set.

23andMe GWAS: Participants were restricted to a set of individuals of European ancestry who fulfilled the classification criteria of either a probability > 97% to be European + Middle Eastern or a probability > 90% to be European, as determined through an analysis of local ancestry. A maximal set of unrelated individuals was chosen for each analysis using a segmental identity-by-descent (IBD) estimation algorithm. Individuals were defined as related if they shared more than 700 cM IBD, including regions where the two individuals shared either one or both genomic segments IBD. SNPs with $P_{HWE} \leq 1 \times 10^{-20}$, a call rate < 95%, or with large allele frequency discrepancies compared to European 1000Genomes reference data were excluded.

Heritability analysis

In LDSC-based heritability analysis, because very large effect sizes can increase LDSC regression standard errors, we chose a two-step estimator via flag --two-step 80. For LDAK-based analyses, heritability models, including GCTA/LDSC, LDAK, hybrid and BLD-LDAK, were specified according to the guidance of the calc-tagging module. The SumHer module was used to calculate heritability using LDAK, hybrid and BLD-LDAK models, where multiplicative inflation and additive inflation were controlled by setting the flags --genomic-control and --intercept both to YES. Additional features included in BLD-LDAK and BLD-LDAK+alpha models followed the recommendations provided by the LDAK developers (https://dougspeed.com/technical-details/). In brief, the BLD-LDAK model includes 64 annotations from the Baseline LD model of LDSC, the LDAK LD weightings, and scaling annotations based on MAF. For BLD-LDAK+alpha, a further parameter is added to allow *alpha* to vary.

Prevalence estimates of RLS in European ancestry populations were chosen based on published studies.[3,4]

Genetic correlation analysis: Sources and filtering criteria for selection of GWAS summary statistics

We extracted summary statistics for diverse traits and diseases from the University of Bristol Integrative Epidemiology Unit OpenGWAS server https://gwas.mrcieu.ac.uk), cross-referencing with GWAS atlas (https://atlas.ctglab.nl/) and augmenting with our own resources (primarily for GWAS of iron and blood cell traits). The following criteria were used to exclude studies of low power, non-matching ancestry, or low quality:

1. Exclude studies with non-European ancestry participants contributing to the summary statistics (even if the number of non-European participants is low or mixed models were used to account for population stratification).
2. Exclude studies with total n < 5.000.
3. For case-control studies (binary traits), exclude studies with < 1,000 cases.
4. Based on recommendations by LDSC authors and GWAS Atlas, remove traits with a heritability Z-score < 2.
5. Based on recommendations by LDSC authors and GWAS Atlas, remove traits with total SNP number < 450,000.
6. For traits with similar phenotype definition (as given in the GWAS atlas file or assumed based on the fact that the data are from the same consortium), exclude the smaller study. However, as study sample size and SNP number both play a role – only use smaller study if SNP number of larger study is lower (compared to smaller study as well as to average SNP number across all studies which, for the studies left after filtering steps 1-3, is 8.7×10^6 SNPs, max 34×10^6, min 20,000; median 9.2×10^6).

Gene prioritization in risk loci

For all LD calculations, the European ancestry 1000 Genomes Phase 3 data was used as the reference panel.

DEPICT: We ran DEPICT (v1.rel194) to first map SNPs to independent loci using the clumping algorithm in PLINK as described above and then performed gene prioritization in these loci based on DEPICT's built-in eQTL data and reconstituted gene sets.[5] Genes with an FDR < 0.05 were considered as significant and prioritized.

FUMA: We used the FUMA web platform (https://fuma.ctglab.nl/, v1.3.6a) for three prioritization schemes to annotate genes in genomic risk loci:[6]

1) Positional mapping with the maximum distance between SNP and gene set at 10 kb.
2) eQTL mapping using the built-in eQTL datasets,limiting the set of candidate eQTLs to significant SNP-gene pairs only (FDR < 0.05).
3) Topology-based mapping using the built-in chromatin interaction data, limited to interactions with an FDR < $1x10^{-5}$ only and defining the promoter region window within 250 bp upstream and 500 bp downstream of the transcription start site.

We ran analyses 2) and 3) once including data from all tissues and once limited to CNS-related studies.

MAGMA: We used MAGMA v1.08 to perform a gene-level GWAS via the test of mean SNP association.[7] Gene locations were based on the NCBI 37.3 annotation provided for MAGMA (https://ctg.cncr.nl/software/magma). We mapped a SNP to a gene if it resided within the gene boundaries or 5 kb of either endpoint. Genes with an FDR < 0.05 were considered as significant and prioritized.

Transcriptome-based association study: We performed genome-wide expression-based gene mapping using S-PrediXcan followed by S-MultiXcan (MetaXcan package v0.7.4) with GTEx-v8 data (https://github.com/hakyimlab/MetaXcan).[8] We used MASHR (multivariate adaptive shrinkage in R) models which account for recombination rate and LD structure. Cross-tissue analysis was performed with S-MulTiXcan and included S-PrediXcan results from all 13 CNS tissues available in GTEx. Significance threshold was set at FDR < 0.05.

Colocalization analysis: We used eCAVIAR (v2.2, https://github.com/fhormoz/caviar) to assess colocalization for each locus–tissue pair using GTEx v8 CNS tissues and their respective significant eQTL SNPs (FDR < 0.05).[9] A maximum of 5 causal SNPs per locus was set. Any eGene with a colocalization posterior probability > 0.1 was prioritized as a target gene.

Fine-mapping of putative causal variants in risk loci: Statistical fine-mapping was performed with CAVIARBF (v0.2.1, https://bitbucket.org/Wenan/caviarbf/src/master/). We used the 74 baseline annotations as available in stratified LD score regression (https://alkesgroup.broadinstitute.org/LDSCORE/).[10] SNPs within 50 kb of a lead SNP and with a MAF > 0.01 were considered. The subset of Europeans in the 1000 Genomes Phase 3 reference panel was used to estimate the LD matrix and 0.2 was added to the diagonal as recommend for reference panel-based estimation with CAVIARBF. The exact Bayes factor was averaged over prior variances of 0.01, 0.1, and 0.5. The elastic net parameters were selected via 10-fold cross-validation.

Colocalization analysis for SLC40A1

For iron-related traits and the *SLC40A1* gene, the sharing of a causal variant was assessed by colocalization analysis with the coloc R package v5.1.0.[11] The coloc.abf function (default priors) was used to test for sharing a causal variant at this locus under five different hypotheses: H0: neither trait has a genetic association in the region, H1: only trait 1 has a genetic association in the region, H2: only trait 2 has a genetic association in the region, H3: both traits are associated, but with different causal variants, H4: both traits are associated and share a single causal variant. Posterior probabilities greater than 90% were considered as suggestive evidence for the respective hypothesis. For this analysis, we used summary statistics of GWASs on QSM and T2* brain MRI measures available from UK Biobank to assess brain iron content as well as summary statistics for peripheral iron measures (ferritin, total iron binding capacity (TIBC), transferrin saturation (TSAT), and serum iron) from a recent meta-analysis (summary stats used were those excluding the INTERVAL study).[12-15]

Tissue and cell-type enrichment analyses

Input datasets: For tissue-level validation with CELLECT, we used the GTEx V8 gene read counts (https://gtexportal.org/home/downloads/adult-gtex/bulk_tissue_expression).

For cell-type level enrichment, we used the adult/adolescent and developmental mouse CNS single-cell RNAseq datasets provided on mousebrain.org (level 1 and level 2 annotations).[16,17] Human datasets comprised the GSEA MSigDb cell type signature gene sets (MSigDb v7.4, human collection C8), the DESCARTES human fetal tissue single cell RNAseq dataset, and the BrainSpan developmental transcriptome.[18-21] For the latter two human datasets, we could compute ES scores and analyze the data with CELLEX and CELLECT.

Simulation study for gene-environment interaction

The properties of LDSC with omitted covariates are discussed in a simulation study by Omer et al.[22] Their results show that 1) LDSC remains accurate for genetic correlation with omitted covariates, 2) the neglect of binary covariates causes heritability estimates to be biased upward. This scenario appears to match the situation of the sex-specific RLS results. Therefore, an important binary covariate may have been omitted in our female GWAS analysis.

To model such an influence, we calculate the liability risk level φ, in N individuals with M SNPs as:

$$\varphi = g_0 + g_1 \circ E + \tau E + \epsilon = X\beta + X\eta \circ E + \tau E + \epsilon \quad \text{(a.1)}$$

Where g_0 is the genetic effect, g_1 is genetic effect interacting with an environmental factor, E is an N vector of the normalized environmental factor, τ is effect size of E, X is the $N \times M$ normalized genotype matrix, β and η are M vectors for the genotypic and interaction effect sizes, respectively, ϵ is the random residual, and $\circ$ represents the Hadamard product.

According to the central moment theory of standard normal distribution of two independent random variables A and B:

$$Cov[AB, A] = \mathbb{E}[A^2B] - \mathbb{E}[[\![A]\!]]^2\mathbb{E}[B] = Var[A]\mathbb{E}[B] = 0$$

Therefore, given X and E are independent:

$$Var[\varphi] = Var[g_0] + Var[g_1E] + Var[\tau E] + Var[\epsilon] \quad \text{(a.2)}$$

When there is no interaction, $\eta = 0$, and E is not specified, the model reduces to the standard form, where:

$$\varphi = X\beta + \epsilon \quad \text{(a.3)}$$

with $E[\epsilon] = 0$, $Var[\epsilon] = (1 - h^2)$, $E[\beta] = 0$, $Var[g_0] = h^2$, and

$$E[\varepsilon] = 0,\ Var[\varepsilon] = (1 - h^2),\ E[\beta] = 0,\ Var[g_0] = h^2, \text{ and}$$

$$E[X_j^2] = N \times Var[\beta_i] = Nh^2 l_j/M + 1 = \frac{Nh^2}{M_{eff}} + 1$$

The expectation of X_2-statistics is approximately equal for both linear and logistic regression at large sample sizes.

Now assume that the true phenotype is given as, $\varphi' = X\beta + X \circ E + \varepsilon$, which includes a gene-environment interaction term. Thus, $\beta_j' = \beta_j + \eta_j\omega$, where ω is a random coefficient following the sampling distribution of E, and $Var(\beta_j') = Var(\beta_j) + Var(\eta\omega)$. In the model (a.3), the heritability then inadvertently depends on the variance of the interaction term, so that sex-dependency of E results in sex-dependency of the h^2, while the GWAS result would not reflect a sex-differential interaction. In an alternative hypothetical scenario, the difference of heritability is contributed by the size of the genetic effect, $\varphi' = kX\beta + \varepsilon$, where k is the ratio of the effect size between two sexes with $\beta_j' = k\beta_j$ and $Var(\beta') = k^2Var(\beta)$. This scenario would also imply sex-dependency of the heritability h^2, as described in Lee's R^2.[23] The k would have to be interpreted as a dosage compensation coefficient. However, unlike the case of the X chromosome, there is no molecular mechanistic basis for assuming a sex-dependent dose effect overarching all autosomes. Hence, we set out to evaluate different scenarios of environmental influence being omitted in the GWAS to check whether they can account for the observed sex-difference in heritability despite similar additive genetic effect sizes in both sexes. Since logistic regression is not readily accessible for analytic examination, we performed a simulation study where we generated different scenarios of environmental effects *E* with or without sex-specific gene-environment interaction, and then checked which of these fitted best our GWAS results.

In detail, we performed the following data simulation steps:

1) Assume that the two sexes have the same additive heritability h^2, if all environmental effects have been removed.

2) Generate the random effect ε, a vector from a normal distribution with $E(\epsilon) = 0$ and $Var(\epsilon) = 1 - h^2$.

3) Generate normalized genotypic effect X via random sampling M SNPs based PRS, where M =193, i.e., the number of lead SNPs in the pooled GWAS.

4) Generate the additive genetic effect $G = X\beta$, where β corresponds to the effect sizes of the pooled GWAS so that $Var(G) = h^2$.

5) Obtain liability risk $\varphi = G + \varepsilon$ as the base model.

6) Obtain disease status $o = I\varphi > \varphi_{cutoff}$ with $\varphi_{cutoff} = \Phi^{-1}(1 - prev)$ where I is the indicator function, Φ^{-1} is the inverse of the standard normal cumulative distribution function, and prev = 0.06 is the empirical European RLS prevalence.

7) Simulate a random vector E and environmental effects η and τ as unobserved additive and interactive environmental effects. E follows a normal distribution (when simulating a continuous covariate scenario) or a Bernoulli distribution (binary covariate scenario) with probability p=1 - $p_{childlessness}$, where $p_{childlessness}$ = 0.21 is the proportion of females without children.

8) For optimized (see below) E, η, and τ, obtain liability risk $\varphi' = X\beta + X \circ E + \tau E + \varepsilon$ with $X\beta$ as in (4), $E(\varepsilon) = 0$, and $Var(\varepsilon) = 1 - (Var(X\beta) + Var(X\eta \circ E) + Var(\tau E))$ in accordance with eq(a.2).

9) Obtain disease status $o' = I\varphi' > \varphi_{cutoff})$ as in (6) with $\varphi'_{cutoff} = \varphi_{cutoff}$, assuming that the liability threshold of the disease is independent of environmental influence.

10) For the simulations in (5,6) and (8,9) estimate E-omitting GWAS summary statistics $\hat{\beta}$, $\widehat{\beta'}$, $\hat{z}$ and $\widehat{z'}$ with models $logit(P(o))$~X and $logit(P(o'))$~X, respectively.

11) Calculate $\gamma_b = \hat{\beta}/\widehat{\beta'}$, representing the mean male-to-female effect size ratio (coefficient).

12) Calculate liability scaled h^2 and h'^2, representing males and females, respectively.

Overall, we simulated four scenarios, including continuous or discrete environment factors, and with or without the inclusion of interactions.

Based on the real GWAS summary statistics, the actually observed heritabilities and mean male-to-female effect size ratio, $h_m^2 \sim N(0.13, 0.014)$, $h_f^2 \sim N(0.32, 0.029)$, and

$\beta_{obs_m}/\beta_{obs_f} \sim N(0.91, 0{,}12)$, were used to find an optimal selection of scenario, E, η, and τ by an adaptive MCMC method, DRAM within the R package BayesianTools v0.0.10 (https://github.com/florianhartig/BayesianTools).

Risk prediction: Creation of a synthetic population for prediction evaluation

The GWAS study based on case-control design aims to estimate genetic effects. The artificial selection bias in the experiment, such as population stratification, sex, and age, was added to the model in the form of covariates and eliminated. Under the conditional independence assumption, PRS can be used to predict the genetic risk of disease. On the other hand, the risk of RLS is closely related to many factors such as sex, age, and obesity. In real-world practice, ignoring these factors may cause a loss of predictive power. However, due to selection bias, the effects of these factors as well as potential interaction effects cannot be estimated from the case-control design.

To better predict the risk of RLS and evaluate the contribution of genetic factors, we created a synthetic population as simplified microscopic representation of the actual population. The synthetic population is restricted to meet the sex and age distribution of the demographic data for Germany as aggregate-level information.

Assuming that the collection of patients in the EU-RLS-GENE dataset is unbiased, the incidence can be estimated based on Bayes' theorem. Following the denomination of survival analysis, the event rate at age a is

$$h(a) = -\frac{S'(a)}{S(a)} \qquad (1)$$

where $S(a)$ is the survival function and $S'(a)$ is the survival event density. To accommodate the sampling strategy for our dataset, we use the sampling probability to represent the hazard function. As for an individual i, the event rate at age a is

$$p_i(rls = 1|age = a) = h(a) \qquad (2)$$

the age distribution at age a is

$$p_i(age = a) = g(a) \qquad (3)$$

In the cohort data, $g(a)$ is the survival density function $S'(a)$. Whereas in our data, it does not vary with a, if the retrospective process is synchronous, such as not affected by memory or disease progression (assumption A1),

$$p_i^y(age = a) = 1/y \qquad (A1)$$

where y is the age of i when the individual is collected. The cumulative risk for the collected individual i is

$$p_i^y(rls = 1) = \sum_{a=1}^{y} p_i^y(age = a, rls = 1)$$

$$= \sum_{a=1}^{y} p_i^y(rls = 1|age = a) \cdot g^y(a)$$

$$= \sum_{a=1}^{y} h(a) g^y(a) \qquad (4)$$

According to Bayes' theorem, for individual i, who currently or previously suffered from RLS, the distribution of age at onset is

$$p_i^y(age = a|rls = 1) = h(a) \cdot g(a, y) / \sum_{t=1}^{y} h(a) g^y(a) \ (5)$$

which could be estimated by empirical distribution function,

$$\widehat{p_i^y}(age = a|rls = 1) = n_a^y / N^y \qquad (6)$$

where n_a^y is the number of cases with age y at collection and age a at onset, N^y is the total number. Combining equations (5) and (6), we have the estimated hazard function as

$$\hat{h}(a) = \frac{n_a^y}{N^y} \cdot \frac{\sum_{a=1}^{y} h(a) g^y(a)}{g^y(a)} \qquad (7)$$

Thus, we have the relative risk ratio as

$$\frac{\hat{h}(j)}{\hat{h}(k)} = \frac{n_j^y}{n_k^y} \cdot \frac{g^y(k)}{g^y(j)} \qquad (8)$$

Given assumption (A1),

$$\frac{\hat{h}(j)}{\hat{h}(k)} = \frac{n_j^y}{n_k^y} \qquad (9)$$

Thus, for age at collection y, h_a is proportional to n_a^y in the case group. To sum up the risk proportions over all different ages Y at collection, we use weighted sum relative risk ratio matrix,

$$\hat{R}_{j,k} = \frac{\hat{h}(j)}{\hat{h}(k)} = \sum_y^Y \frac{n_j^y + n_k^y}{N_j + N_k} (n_j^y / n_k^y) \qquad (10)$$

where N_j and N_k is the total number of cases with age at onset j or k.

Based on the relative risk ratio matrix, the age dependent risks were solved by maximum likelihood estimation (MLE). The estimated age dependent risks were expected to produce a

relative risk matrix that will maximize the similarity to the estimated $\hat{R}$ (eq 10) shown above. The log-likelihood function is

$$L(\hat{h}, \hat{R}) = -\sum_{j>k} \log\left(\frac{1}{\sqrt{2\pi}}\, e^{-\frac{1}{2}(\hat{h}_j/\hat{h}_k - \hat{R}_{j,k})^2}\right) \quad (11)$$

The MLE was solved by a quasi-Newton method, BFGS in R package stats4 v4.1.0. Without loss of generality, $\hat{h}_{a=10}$ is set to 1, and the solved r_a is equal to the absolute value $\hat{h}_a$ divided by $\hat{h}_{a=10}$.

To estimate the absolute $\hat{h}$, the scale factor s can be estimated by fitting to the prevalence,

$p_{2012}(RLS = 1) = 10\%$

$$= \sum_{y=1}^{Y}\left(p_{2012}(age = y) \cdot \sum_{a=1}^{y} s \cdot r_a\right),$$

where $p_{2012} = (age = y)$ is the population age distribution of Germany in 2012.

Following the method shown above, age-dependent risks were estimated for males and females separately. Distributions of ages and sexes in Germany were based on World Population Prospects of United Nations, Department of Economic and Social Affairs, and were downloaded from https://www.populationpyramid.net/germany.

In the previous step of fitting the prevalence, the proportion of cases and controls for each sex in each age group was derived. The synthetic population is generated by resampling our GWAS dataset to match these sex and age distributions. Besides, for each resampled case, we prioritize the matched controls that have the most similar population stratification, which was measured by the Euclidean distance of the top 10 MDS.

For the evaluation dataset from INTERVAL study, we assume that the data were collected from the same cohort and the age at onset information was obtained in the questionnaire for all people. The matched case/control dataset were reconstructed with following procedure:

```
For each i in ages:
  [incidence at i] = [number of cases with aao = i] /
                     ([number of cases with aao >= i] +
                      [number of controls with age >= i])
  [prevalence at i] = [sum of incidences from 0 to i]
  [expected number of control with age at i ] =
                    (1-[prevalence at i] / [prevalence at i]) *
                     [number of cases with aao = i]
  resampling {control with age at i} with replacement to [expected number of control with age at i ]
  resampling {cases with aao at i} with replacement to [number of cases with aao = i]
```

To avoid data leakage, the data were first randomly split into two parts, 50% for calibration and 50% for testing, before establishing case/control matches. In the procedure, the resampling with replacement was repeated 10 times for bootstrap-based error estimation. ages were split to 7 bins in between the ages 0,20,30,40,50,60,70,70+.

URLs for software

PLINK (v1.90b6.7): https://www.cog-genomics.org/plink/1.9/

SNPTEST (v2.5.4) https://www.chg.ox.ac.uk/~gav/snptest/

SAIGE (0.35.8.8): https://github.com/saigegit/SAIGE

N-GWAMA (v1.2.6) https://github.com/baselmans/multivariate_GWAMA;

METAL (release 2011-03-25): https://csg.sph.umich.edu/abecasis/metal/index.html

METASOFT (v2.0.1): https://web.cs.ucla.edu/~eeskin/

GCTA (v1.93.0beta): https://yanglab.westlake.edu.cn/software/gcta/#Overview

LDSC (v1.0.1): https://github.com/bulik/ldsc

LDAK (v5.0): https://dougspeed.com/ldak/

LHC-MR (v0.0.0.9000): https://github.com/LizaDarrous/lhcMR

DEPICT (v1 rel194): https://github.com/perslab/depict

FUMA (v1.3.6a): https://fuma.ctglab.nl/

MAGMA (v1.08) https://cncr.nl/research/magma/

MetaXcan (v0.7.4) https://github.com/hakyimlab/MetaXcan

eCAVIAR (v2.2): https://github.com/fhormoz/caviar

CAVIARBF (v0.2.1): https://bitbucket.org/Wenan/caviarbf/src/master/

CELLECT (v1.3.0) and CELLEX (v1.2.1): https://github.com/perslab/CELLECT

MAGMA_celltyping (v2.0.0): https://github.com/neurogenomics/MAGMA_Celltyping

pycox (v0.2.1): https://github.com/havakv/pycox

PyTorch (v1.6.0): https://github.com/pytorch/pytorch

H2O autoML (v3.36.0.2): https://docs.h2o.ai/h2o/latest-stable/h2o-docs/automl.html

Sanger imputation server: https://imputation.sanger.ac.uk/

EAGLE2 (v2.0.5): https://alkesgroup.broadinstitute.org/Eagle/

PBWT (v3.1): https://github.com/richarddurbin/pbwt

Minimac3: https://genome.sph.umich.edu/wiki/Minimac3

R (v4.0.4 and v4.0.2): https://cran.r-project.org/

Rrvgo package (v1.2.0):https://bioconductor.org/packages/release/bioc/html/rrvgo.html

WGCNA package (v1.69): https://cran.r-project.org/web/packages/WGCNA/index.html

TwoSampleMR package (v0.5.6): https://mrcieu.github.io/TwoSampleMR/index.html

Coloc package (v5.1.0) https://chr1swallace.github.io/coloc/

BayesianTools package (v0.0.10): https://github.com/florianhartig/BayesianTools

Bigsnpr package (v1.12.2), implements LDpred2: https://privefl.github.io/bigsnpr/

gwasvcf package (v0.1.0): https://github.com/MRCIEU/gwasvcf

randomForestSRC package (v3.0.1): https://www.randomforestsrc.org/

References

1. Allen, R.P. *et al.* Restless legs syndrome/Willis-Ekbom disease diagnostic criteria: updated International Restless Legs Syndrome Study Group (IRLSSG) consensus criteria--history, rationale, description, and significance. *Sleep Med* **15**, 860-73 (2014).
2. Jun, G. *et al.* Detecting and estimating contamination of human DNA samples in sequencing and array-based genotype data. *Am J Hum Genet* **91**, 839-48 (2012).
3. Innes, K.E., Selfe, T.K. & Agarwal, P. Prevalence of restless legs syndrome in North American and Western European populations: a systematic review. *Sleep Med* **12**, 623-34 (2011).
4. Ohayon, M.M., O'Hara, R. & Vitiello, M.V. Epidemiology of restless legs syndrome: a synthesis of the literature. *Sleep Med Rev* **16**, 283-95 (2012).
5. Pers, T.H. *et al.* Biological interpretation of genome-wide association studies using predicted gene functions. *Nat Commun* **6**, 5890 (2015).
6. Watanabe, K., Taskesen, E., van Bochoven, A. & Posthuma, D. Functional mapping and annotation of genetic associations with FUMA. *Nat Commun* **8**, 1826 (2017).
7. de Leeuw, C.A., Mooij, J.M., Heskes, T. & Posthuma, D. MAGMA: generalized gene-set analysis of GWAS data. *PLoS Comput Biol* **11**, e1004219 (2015).
8. Barbeira, A.N. *et al.* Exploring the phenotypic consequences of tissue specific gene expression variation inferred from GWAS summary statistics. *Nat Commun* **9**, 1825 (2018).
9. Hormozdiari, F. *et al.* Colocalization of GWAS and eQTL Signals Detects Target Genes. *Am J Hum Genet* **99**, 1245-1260 (2016).
10. Finucane, H.K. *et al.* Partitioning heritability by functional annotation using genome-wide association summary statistics. *Nat Genet* **47**, 1228-35 (2015).
11. Wallace, C. A more accurate method for colocalisation analysis allowing for multiple causal variants. *PLoS Genet* **17**, e1009440 (2021).
12. Bell, S. *et al.* A genome-wide meta-analysis yields 46 new loci associating with biomarkers of iron homeostasis. *Commun Biol* **4**, 156 (2021).
13. Wang, C. *et al.* Phenotypic and genetic associations of quantitative magnetic susceptibility in UK Biobank brain imaging. *Nat Neurosci* **25**, 818-831 (2022).
14. Smith, S.M. *et al.* An expanded set of genome-wide association studies of brain imaging phenotypes in UK Biobank. *Nat Neurosci* **24**, 737-745 (2021).
15. Elliott, L.T. *et al.* Genome-wide association studies of brain imaging phenotypes in UK Biobank. *Nature* **562**, 210-216 (2018).
16. La Manno, G. *et al.* Molecular architecture of the developing mouse brain. *Nature* **596**, 92-96 (2021).
17. Zeisel, A. *et al.* Molecular Architecture of the Mouse Nervous System. *Cell* **174**, 999-1014 e22 (2018).
18. Cao, J. *et al.* A human cell atlas of fetal gene expression. *Science* **370**(2020).
19. Miller, J.A. *et al.* Transcriptional landscape of the prenatal human brain. *Nature* **508**, 199-206 (2014).
20. Liberzon, A. *et al.* Molecular signatures database (MSigDB) 3.0. *Bioinformatics* **27**, 1739-40 (2011).
21. Subramanian, A. *et al.* Gene set enrichment analysis: a knowledge-based approach for interpreting genome-wide expression profiles. *Proc Natl Acad Sci U S A* **102**, 15545-50 (2005).
22. Weissbrod, O., Flint, J. & Rosset, S. Estimating SNP-Based Heritability and Genetic Correlation in Case-Control Studies Directly and with Summary Statistics. *Am J Hum Genet* **103**, 89-99 (2018).
23. Lee, S.H., Goddard, M.E., Wray, N.R. & Visscher, P.M. A better coefficient of determination for genetic profile analysis. *Genet Epidemiol* **36**, 214-24 (2012).

Supplementary Figure 1

Comparison of different cell-type enrichment approaches (MAGMA_celltyping and CELLECT)

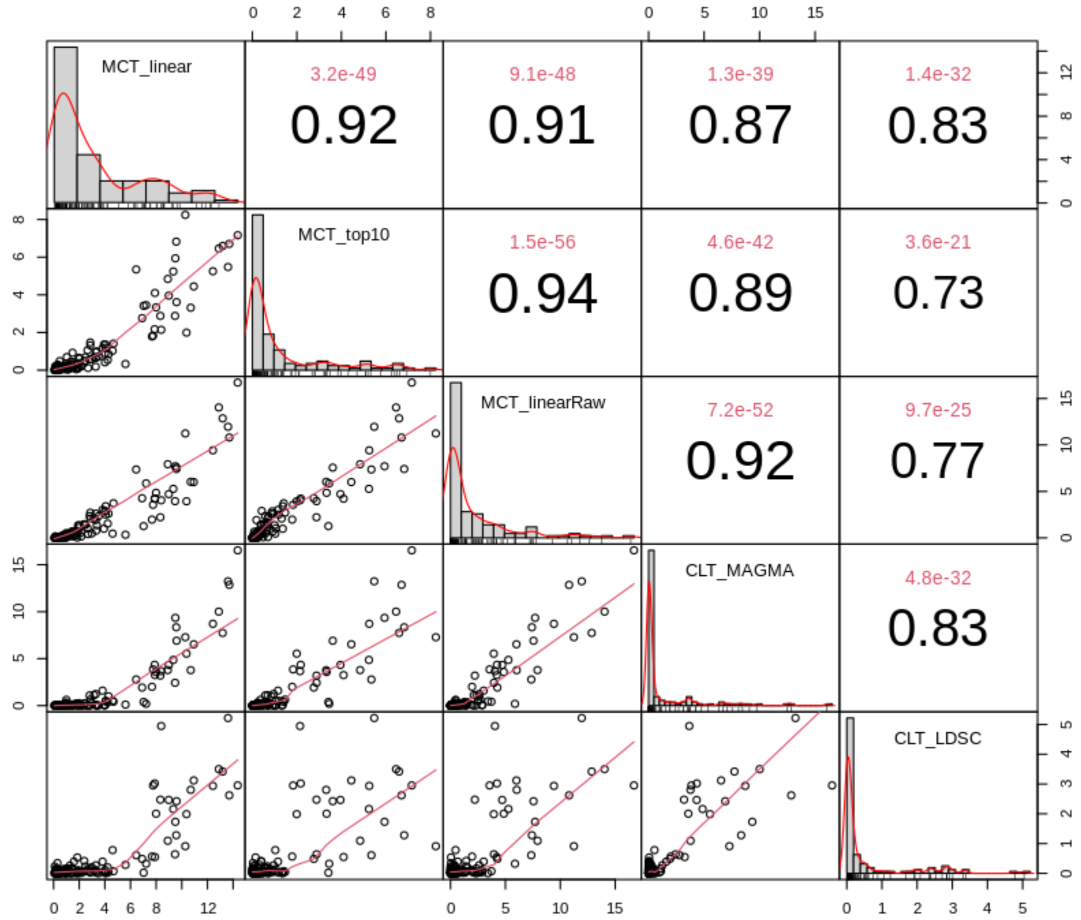

Pairwise correlation chart between prioritization pipelines showing correlation between p-values obtained with the different methods: The x and y-axes show nominal -$\log_{10}$(p-value); MCT_linear stands for MAGMA_Celltyping linear mode, where the specificity score is grouped in 40 quantile bins. MCT_top10 stands for MAGMA_Celltyping top10 mode; MCT_linearRaw stands for MAGMA_Celltyping using raw specificity score (EWCE); lower-diagonal cells show scatter plot and local regression (lowess)-based fitted line; upper-diagonal cells show Pearsons's correlation coefficient with the corresponding p-value (one-sample two-sided Z-test). Diagonal cells show a histogram of the -$\log_{10}$(p-value) obtained with each pipeline (one-sided tests). We ran MAGMA_celltyping in its linear as well as its top10% enrichment mode and ES scores calculated with EWCE as recommended (https://github.com/neurogenomics/MAGMA_Celltyping). The analyses were performed using the level 2 mousebrain developmental dataset (http://mousebrain.org/development/).[16]

Consortium membership information

Complete member list of D.E.S.I.R. study group

B Balkau, P Ducimetière, E Eschwège (Center for Research in Epidemiology and Population Health (CESP), INSERM U1018, Villejuif, France)
F Rancière (Université Paris Descartes, Paris, France)
F Alhenc-Gelas (INSERM U367, Paris, France)
Y Gallois, A Girault (CHU D'Angers, Angers, France)
F Fumeron, M Marre, L Potier, R Roussel (Université Paris Cité, INSERM UMR-S1151, CNRS UMR-S8253, Institut Necker-Enfants Malades, Paris, France; Department of Diabetology, Endocrinology and Nutrition, Assistance Publique-Hôpitaux de Paris, Bichat Hospital, DHU FIRE, Paris, France)
F Bonnet (CHU de Rennes, Rennes, France)
A Bonnefond, S Cauchi, P Froguel (Inserm U1283, CNRS UMR 8199, European Genomic Institute for Diabetes (EGID), Institut Pasteur de Lille, Lille, France; University of Lille, Lille University Hospital, Lille, France)
J Cogneau (Institute de Recherche Médecine Générale, Paris, France)
C Born, E Caces, M Cailleau, O Lantieri, JG Moreau, F Rakotozafy, J Tichet, S Vol (Institute Inter-Regional pour la Santé, La Riche, France)
General practitioners of the Region
Centres d'Examens de Santé: Alençon, Angers, Blois, Caen, Chateauroux, Chartres, Cholet, Le Mans, Orléans, Tours

Complete member list of 23andMe research team

Michelle Agee, Stella Aslibekyan, Adam Auton, Robert K. Bell, Katarzyna Bryc, Sarah K. Clark, Sarah L. Elson, Kipper Fletez-Brant, Pierre Fontanillas,, Pooja M. Gandhi, Karl Heilbron, Barry Hicks, Karen E. Huber, Ethan M. Jewett, Yunxuan Jiang, Aaron Kleinman, Keng-Han Lin, Nadia K. Litterman, Marie K. Luff, Jey C. McCreight, Matthew H. McIntyre, Kimberly F. McManus, Joanna L. Mountain, Sahar V. Mozaffari, Elizabeth S. Noblin, Carrie A.M. Northover, Jared O'Connell, Aaron A. Petrakovitz, Steven J. Pitts, G. David Poznik, J. Fah Sathirapongsasuti, Anjali J. Shastri, Janie F. Shelton, Suyash Shringarpure, Chao Tian, Joyce Y. Tung, Robert J. Tunney, Vladimir Vacic, Xin Wang, Amir S. Zare, Nicolas A. Furlotte, Priyanka Nandakumar, David A. Hinds.

Complete member list of DBDS Genomic consortium

Karina Banasik (Novo Nordisk Foundation Center for Protein Research, Faculty of Health and Medical Sciences, University of Copenhagen, Copenhagen, Denmark)
Jakob Bay (Department of Clinical Immunology, Zealand University Hospital, Køge, Denmark)
Jens Kjærgaard Boldsen (Department of Clinical Immunology, Aarhus University Hospital, Aarhus, Denmark)
Thorsten Brodersen (Department of Clinical Immunology, Zealand University Hospital, Køge, Denmark)
Søren Brunak (Novo Nordisk Foundation Center for Protein Research, Faculty of Health and Medical Sciences, University of Copenhagen, Copenhagen, Denmark)

Kristoffer Burgdorf (Novo Nordisk Foundation Center for Protein Research, Faculty of Health and Medical Sciences, University of Copenhagen, Copenhagen, Denmark; Department of Clinical Immunology, Copenhagen University Hospital, Rigshospitalet, Copenhagen, Denmark)
Mona Ameri Chalmer (Danish Headache Center, Department of Neurology, Copenhagen University Hospital, Rigshospitalet-Glostrup, Copenhagen, Denmark)
Maria Didriksen (Department of Clinical Immunology, Copenhagen University Hospital, Rigshospitalet, Copenhagen, Denmark)
Khoa Manh Dinh (Department of Clinical Immunology, Aarhus University Hospital, Aarhus, Denmark)
Joseph Dowsett (Department of Clinical Immunology, Copenhagen University Hospital, Rigshospitalet, Copenhagen, Denmark)
Christian Erikstrup (Department of Clinical Immunology, Aarhus University Hospital, Aarhus, Denmark; Department of Clinical Medicine, Health, Aarhus University, Aarhus, Denmark)
Bjarke Feenstra (Department of Clinical Immunology, Copenhagen University Hospital, Rigshospitalet, Copenhagen, Denmark; Statens Serum Institut, Copenhagen, Denmark)
Frank Geller (Department of Clinical Immunology, Copenhagen University Hospital, Rigshospitalet, Copenhagen, Denmark; Statens Serum Institut, Copenhagen, Denmark)
Daniel Gudbjartsson (deCODE Genetics, Reykjavik, Iceland)
Thomas Folkmann Hansen (Danish Headache Center, Department of Neurology, Copenhagen University Hospital, Rigshospitalet-Glostrup, Copenhagen, Denmark)
Lotte Hindhede (Department of Clinical Immunology, Aarhus University Hospital, Aarhus, Denmark)
Henrik Hjalgrim (Danish Cancer Society Research Center, Copenhagen, Denmark; Department of Epidemiology Research, Statens Serum Institut, Copenhagen, Denmark)
Rikke Louise Jacobsen (Department of Clinical Immunology, Copenhagen University Hospital, Rigshospitalet, Copenhagen, Denmark)
Gregor Jemec (Department of Clinical Medicine, Zealand University hospital, Roskilde, Denmark)
Katrine Kaspersen (Department of Clinical Immunology, Aarhus University Hospital, Aarhus, Denmark)
Bertram Dalskov Kjerulff (Department of Clinical Immunology, Aarhus University Hospital, Aarhus, Denmark)
Lisette Kogelman (Danish Headache Center, Department of Neurology, Copenhagen University Hospital, Rigshospitalet-Glostrup, Copenhagen, Denmark)
Margit Anita Hørup Larsen (Department of Clinical Immunology, Copenhagen University Hospital, Rigshospitalet, Copenhagen, Denmark)
Ioannis Louloudis (Novo Nordisk Foundation Center for Protein Research, Faculty of Health and Medical Sciences, University of Copenhagen, Copenhagen, Denmark)
Agnete Lundgaard (Novo Nordisk Foundation Center for Protein Research, Faculty of Health and Medical Sciences, University of Copenhagen, Copenhagen, Denmark)
Susan Mikkelsen (Department of Clinical Immunology, Aarhus University Hospital, Aarhus, Denmark)
Christina Mikkelsen (Department of Clinical Immunology, Copenhagen University Hospital, Rigshospitalet, Copenhagen, Denmark)
Kaspar Rene Nielsen (Department of Clinical Immunology, Aalborg University Hospital, Aalborg, Denmark; Department of Clinical Medicine, Aalborg University, Aalborg, Denmark)

Ioanna Nissen (Department of Clinical Immunology, Copenhagen University Hospital, Rigshospitalet, Copenhagen, Denmark)
Mette Nyegaard (Department of Health Science and Technology, Faculty of Medicine, Aalborg Univeristy, Aalborg, Denmark)
Sisse Rye Ostrowski (Department of Clinical Immunology, Copenhagen University Hospital, Rigshospitalet, Copenhagen, Denmark; Department of Clinical Medicine, Faculty of Health and Medical Sciences, University of Copenhagen, Copenhagen, Denmark)
Ole Birger Pedersen (Department of Clinical Immunology, Zealand University Hospital, Køge, Denmark; Department of Clinical Medicine, Faculty of Health and Medical Sciences, University of Copenhagen, Copenhagen, Denmark)
Alexander Pil Henriksen (Novo Nordisk Foundation Center for Protein Research, Faculty of Health and Medical Sciences, University of Copenhagen, Copenhagen, Denmark)
Palle Duun Rohde (Department of Health Science and Technology, Faculty of Medicine, Aalborg Univeristy, Aalborg, Denmark)
Klaus Rostgaard (Danish Cancer Society, Copenhagen, Denmark; Department of Epidemiology Research, Statens Serum Institut, Copenhagen, Denmark)
Michael Schwinn (Department of Clinical Immunology, Copenhagen University Hospital, Rigshospitalet, Copenhagen, Denmark)
Kari Stefansson (deCODE Genetics, Reykjavik, Iceland)
Hreinn Stefánsson (deCODE Genetics, Reykjavik, Iceland)
Erik Sørensen (Department of Clinical Immunology, Copenhagen University Hospital, Rigshospitalet, Copenhagen, Denmark)
Unnur Þorsteinsdóttir (deCODE Genetics, Reykjavik, Iceland)
Lise Wegner Thørner (Department of Clinical Immunology, Copenhagen University Hospital, Rigshospitalet, Copenhagen, Denmark)
Mie Topholm Bruun (Department of Clinical Immunology, Odense University Hospital, Odense, Denmark)
Henrik Ullum (Statens Serum Institut, Copenhagen, Denmark)
Thomas Werge (Institute of Biological Psychiatry, Mental Health Centre, Sct. Hans, Copenhagen University Hospital, Roskilde, Denmark; Department of Clinical Medicine, Faculty of Health and Medical Sciences, University of Copenhagen, Copenhagen, Denmark)
David Westergaard (Novo Nordisk Foundation Center for Protein Research, Faculty of Health and Medical Sciences, University of Copenhagen, Copenhagen, Denmark)
